# Classification of node-positive melanomas into prognostic subgroups using keratin, immune, and melanogenesis expression patterns

**DOI:** 10.1038/s41388-021-01665-0

**Published:** 2021-02-09

**Authors:** Dvir Netanely, Stav Leibou, Roma Parikh, Neta Stern, Hananya Vaknine, Ronen Brenner, Sarah Amar, Rivi Haiat Factor, Tomer Perluk, Jacob Frand, Eran Nizri, Dov Hershkovitz, Valentina Zemser-Werner, Carmit Levy, Ron Shamir

**Affiliations:** 1 Blavatnik School of Computer Science, Tel Aviv University, Tel Aviv, Israel; 2 Sackler Faculty of Medicine, Tel Aviv University, Tel Aviv, Israel; 3 Department of Oncology, Edith Wolfson Medical Center, Holon, Israel; 4 Department of Plastic and Reconstructive Surgery, Edith Wolfson Medical Center, Holon, Israel; 5 Department of Surgery A, Tel Aviv Sourasky Medical Center, Tel Aviv, Israel; 6 Institute of Pathology, Tel Aviv Sourasky Medical Center, Tel Aviv, Israel

**Keywords:** Cancer genomics, Melanoma, Tumour biomarkers

## Abstract

Cutaneous melanoma tumors are heterogeneous and show diverse responses to treatment. Identification of robust molecular biomarkers for classifying melanoma tumors into clinically distinct and homogenous subtypes is crucial for improving the diagnosis and treatment of the disease. In this study, we present a classification of melanoma tumors into four subtypes with different survival profiles based on three distinct gene expression signatures: keratin, immune, and melanogenesis. The melanogenesis expression pattern includes several genes that are characteristic of the melanosome organelle and correlates with worse survival, suggesting the involvement of melanosomes in melanoma aggression. We experimentally validated the secretion of melanosomes into surrounding tissues by melanoma tumors, which potentially affects the lethality of metastasis. We propose a simple molecular decision tree classifier for predicting a tumor’s subtype based on representative genes from the three identified signatures. Key predictor genes were experimentally validated on melanoma samples taken from patients with varying survival outcomes. Our three-pattern approach for classifying melanoma tumors can contribute to advancing the understanding of melanoma variability and promote accurate diagnosis, prognostication, and treatment.

## Introduction

Cutaneous melanoma is the most lethal form of skin cancer, showing a continuous rise in worldwide incidence over the past several decades [1–3]. Melanoma tumors develop by uncontrolled proliferation of melanocytes, the pigment-producing cells of the skin [4]. Primary melanoma tumors are regularly localized to the skin and are usually curable by excision when detected early [5]. However, melanoma tumors tend to metastasize rapidly into surrounding tissues and distant organs and are, therefore, considerably more challenging to cure at later stages [6].

Melanoma tumors are heterogeneous and show high diversity in their biological characteristics, metastatic potential, survival risk, and response to treatment [7]. Therefore, the stratification of melanoma tumors into clinically distinct, prognostic subtypes is crucial for accurate diagnosis, treatment guidance, and subtype-specific drug development. For the past 40 years, a clinicopathological system has been used to classify primary melanomas into four major subtypes (superficial spreading, nodular, lentigo maligna, and acral lentiginous) based on clinical and pathological features [8, 9]. Although beneficial for diagnosis, this classification showed limited clinical relevance, especially for prognosis and treatment guidance [8].

With the emergence of high-throughput genomic technologies, several commonly mutated genes that play a central role in melanoma tumorigenesis and metastasis, such as BRAF, NRAS, and NF1, were identified. These findings significantly advanced the understanding of melanoma progression and led to the development of targeted therapies that have improved patient survival [10, 11].

In 2015, The Cancer Genome Atlas (TCGA) reported on a study of 331 melanoma patients using six different high-throughput omic technologies [12]. The study partitioned melanoma tumors (both primary and metastatic) based on the pattern of the most prevalent mutated genes into four subtypes: BRAF, NRAS, NF1, and WT [12]. While this mutation-based classification has proven beneficial for highlighting key potential subtype-specific drug targets, it provides little prognostic value.

The same study also suggested a transcriptomics-based classification, which divided melanoma tumors into three prognostic groups: high-immune, keratin, and MITF-low [12]. The high-immune group showed the best 10-year survival and was characterized by the overexpression of many immune genes. The keratin group contained most of the primary tumors, conferred the worst survival (possibly due to a bias of large primary tumor thickness in the TCGA cohort), and was characterized by overexpression of keratin, pigmentation, and epithelial genes. Lastly, the MITF-low group showed medium survival and was characterized by the underexpression of keratin and pigmentation genes. Interestingly, these three transcriptomic sample groups showed little agreement with the mutation-based groups. Moreover, the samples in the keratin transcriptomic group showed low consistency in their expression profiles and the clinical labels, suggesting the need for a more refined transcriptomic tumor classification.

In this study, we set out to explore whether the transcription-based subtype classification can be improved based on the larger number of 469 melanoma samples currently available from TCGA. We reasoned that the larger dataset might allow for the identification of new prognostic subtypes or improve the characterization of previously described subtypes. We also aimed at identifying a minimal set of informative prognostic biomarkers that can be used to stratify patients into clinically relevant subtypes. Finally, we performed a set of experimental tests on human melanoma specimens to validate our computational discoveries.

## Results

### Unsupervised analysis identifies four distinct melanoma subgroups

**Fig. 1:**
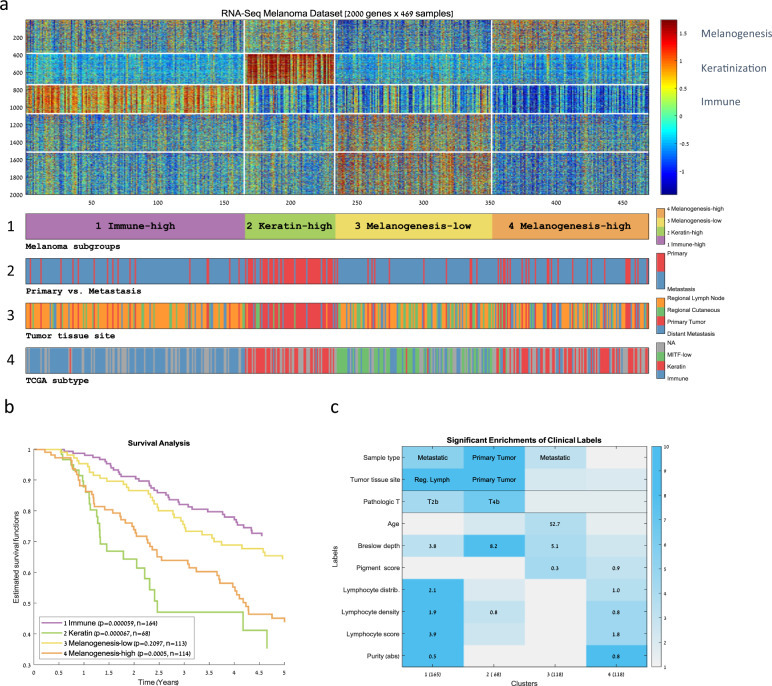
Clustering of TCGA’s RNA-Seq melanoma dataset. **a** A heat map representing the clustering of 469 melanoma samples (matrix columns) into four groups based on the 2000 genes with the most variable expression profiles (matrix rows). Each sample cluster represents a group of similar melanoma tumors. Genes were also clustered in order to identify groups of coexpressed genes. Both samples and genes were clustered using the *k*-means algorithm (using *k* = 4 for the samples and *k* = 5 for the genes). The bars below the matrix display sample labels: (1) cluster ID, (2) primary versus. metastasis, (3) tissue site, and (4) TCGA transcriptomic subtype. **b** Kaplan–Meier curves for the four sample clusters. Log-rank *p* values appear in the legend. **c** Summary of the significant enrichments on sample clusters (columns) for clinical labels (rows). The value within each cell specifies the most significant enrichment based on the hypergeometric distribution. Cells are colored by enrichment significance in −log10 scale.

In order to identify groups of similar melanoma tumors, we applied unsupervised analysis on 469 RNA-Seq expression profiles obtained from TCGA’s melanoma dataset. The dataset contained a mixture of primary (*n* = 104) and metastasis samples (*n* = 365). Clustering of the samples based on the 2000 most variable genes resulted in four distinct sample clusters showing significantly different 5-year survival rates (Fig. 1a, b, and Supplementary Table S1). Gene Ontology (GO) enrichment analysis identified active gene signatures that were used to characterize each sample group (Supplementary Fig. S1). We also used the clinical information available for the samples in order to clinically characterize each sample group (Fig. 1c and Supplementary Fig. S2).

Cluster 2, with the lowest survival rate, was mainly composed of primary melanomas showing significantly high Breslow depths and high pathologic *T* values. This cluster was associated with overexpression of cornification, epidermis development, and keratin-related genes, all of which are characteristic of differentiated keratinocytes that form the outermost skin barrier [13]. We attributed the poor survival in this cluster to the bias in the TCGA cohort for thick primary tumors [12]. The other three clusters were mainly composed of metastatic melanomas. Cluster 1, which conferred the highest survival rate, was enriched for lymph node metastases and showed significantly high values for several immune scores that correlate with lymphocyte infiltration. Cluster 1 was also associated with the overexpression of adaptive immune response genes. Cluster 3 showed relatively good survival and was found to be marginally enriched for regional cutaneous tissue sites, whereas cluster 4 showed relatively poor survival and was found to be marginally enriched for metastasis to distant tissue sites. Interestingly, what distinguished the relatively poor prognosis cluster 4 from the relatively good prognosis cluster 3 was an expression pattern enriched for melanin biosynthesis genes (gene cluster 1) whose overexpression was correlated with poor survival.

We compared our four-cluster partition to TCGA’s three transcriptomic subtypes (Fig. 1a4 and Supplementary Fig. S3). We found that the two partitions largely correspond (Chi-square *p* value = 1.6e−79)—sample clusters 1 and 3 were significantly enriched for TCGA’s immune and MITF-low transcriptomic subtypes, respectively. However, TCGA’s keratin subtype was split into two distinct clusters—the primary-enriched worst outcome cluster 2 and the bad outcome metastasis-enriched cluster 4. Overall, our analysis revealed a partition of the metastatic samples into the high-immune, best survival (cluster 1), low-melanogenesis good survival (cluster 3, corresponding to TCGA’s MITF-low subtypes), and a new metastasis-enriched subgroup, characterized by poor survival and by significant overexpression of melanogenesis genes (cluster 4). We named the four identified melanoma subgroups accordingly: (1) “Immune,” (2) “Keratin,” (3) “Melanogenesis-low,” and (4) “Melanogenesis-high.” Table 1 summarizes the characteristics of the four subgroups and their relation to TCGA’s subgroups.

**Table 1:** Summary of the main sample cluster characteristics.

Cluster	Number of Samples	Cluster name	TCGA transcriptomic subtype enrichment	Survival	Tumor tissue type enrichment	Gene Ontology enrichment of highly expressed genes
1	105	Immune	Immune	Best	Regional lymph node	Immune response
2	68	Keratin	Keratin	Worst	Primary	Cornification
3	118	Melanogenesis-low	MITF-low	Good		Nervous system development
4	118	Melanogenesis-high	Keratin	Bad		Melanin biosynthetic process

### Overexpression of melanogenesis genes characterizes a poor-survival melanoma subtype

In order to further characterize the poor-survival cluster 4 (“Melanogenesis-high”), we analyzed the genes that were overexpressed in this cluster (gene cluster 1). They were enriched for genes related to the synthesis of the melanin pigment (“melanin biosynthetic process,” *p* value < 3.46E−08, see Supplementary Fig. S1). Additional gene sets significantly enriched in that gene cluster were the “Melanogenesis” KEGG-pathway (*p* value < 0.005, nine genes: GNAO1, DCT, KIT, TYRP1, FZD9, ADCY2, ADCY1, TYR, WNT4) and the “Melanosome membrane” GO term (*p* value < 0.0004, six genes: OCA2, SLC45A2, GPR143, DCT, TYRP1, TYR). See Supplementary Tables S2 and S3 for the complete enrichment results.

Interestingly, these results suggest that the “Melanogenesis-high” samples differ from the “Melanogenesis-low” samples by overexpression of genes that are specific to the melanosome organelle (see Supplementary Fig. S4). The melanosome organelle is the hallmark of melanocytes, the melanoma cell of origin [14]. In normal skin, melanosomes are responsible for melanin production, storage, and transport from melanocytes to surrounding keratinocytes [15, 16]. However, the reason melanoma cells retain this function of their cell of origin and the function of the melanosome itself in melanoma cells have only recently begun to be revealed [17, 18]. The melanin biosynthesis genes OCA2, TYRP1, DCT, and PMEL (SILV) also appeared among the top genes overexpressed in “Melanogenesis-high” samples in comparison to all other samples (Supplementary Table S4).

**Fig. 2:**
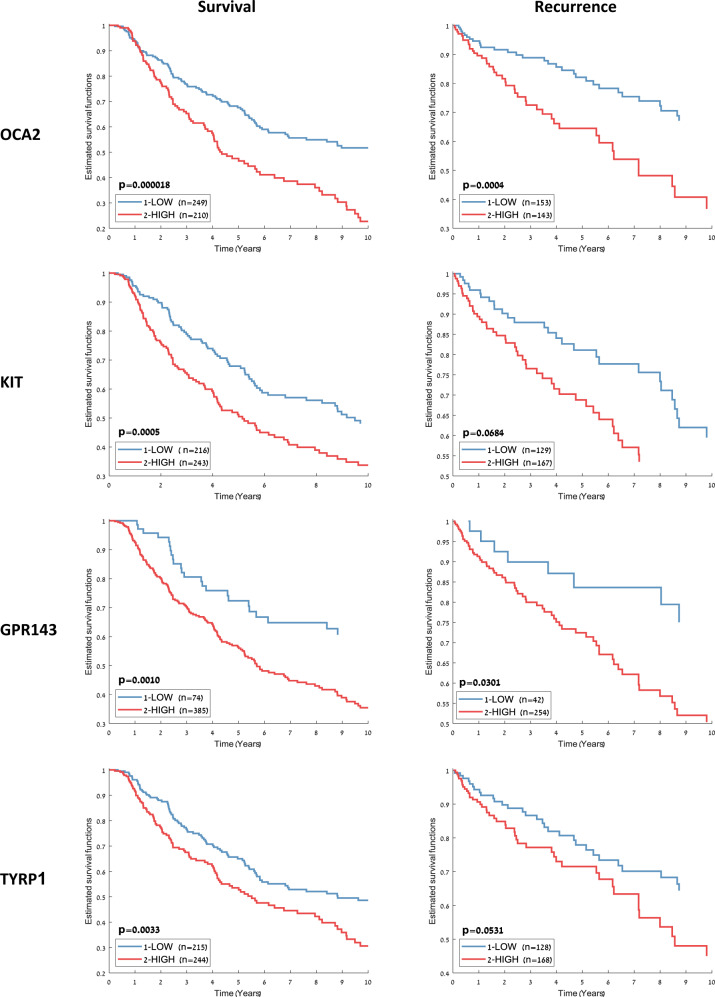
Ten-year survival and recurrence risk estimates for melanosome-related genes. All dataset samples were split into two groups based on the gene expression levels of several melanosome-related genes. For each gene, the threshold for splitting the samples into two groups was the mean of its 5th and 95th expression percentile.

To test the independent prognostic value of those melanosome-related genes, we partitioned all of the dataset samples into two groups based on the expression levels of each gene and calculated the difference in the survival plots of the two groups using the log-rank test. For OCA2, KIT, GPR143, and TYRP1, overexpression was significantly correlated with poorer 10-year survival as well as increased recurrence risk (Fig. 2). These results may suggest a mechanistic link between the melanosome organelle and the increased lethality of melanoma.

### Metastatic melanomas retain the ability to secrete melanosomes into surrounding tissue

**Fig. 3:**
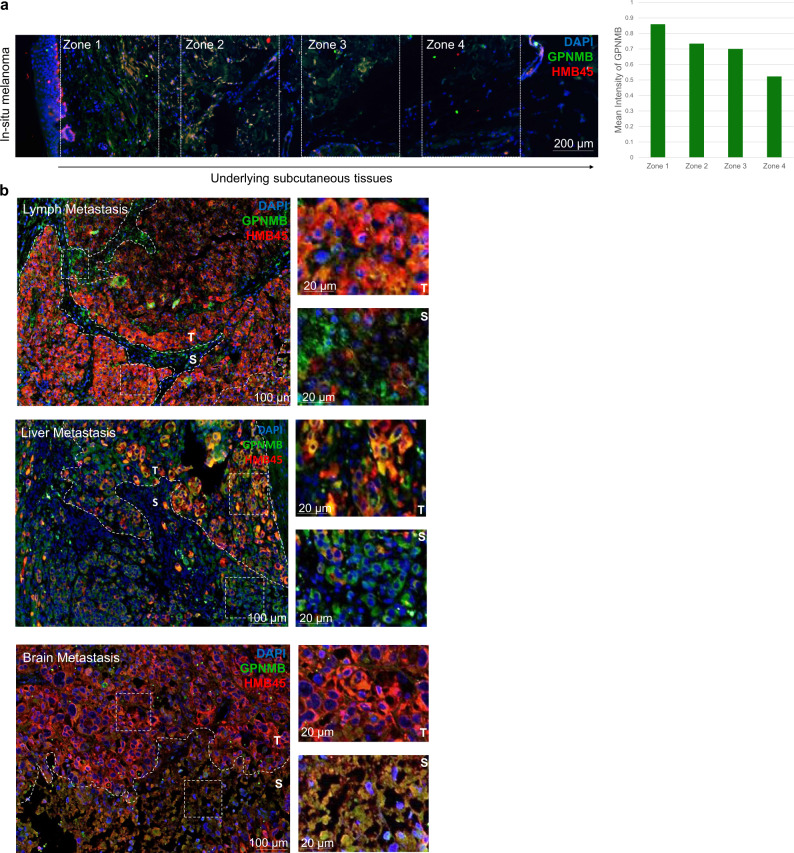
Melanosomes diffuse outward from primary and metastatic tissues. **a** Immunohistochemical (IHC) analysis of an in situ melanoma showing mature melanosomes stained with anti-GPNMB (green) diffusing rightward into the underlying subcutaneous tissues and away from the primary melanoma tumor. HMB45 (red), an antibody for PMEL, which stains the premelanosome, shows the location of the melanoma. Nuclei were stained blue with DAPI. Equally sized, equidistant zones were delineated on the image in order to quantify differences in the intensity of GPNMB displayed by the graph to the right of the image. **b** IHC investigation of the metastatic sites: lymph node (top), liver (middle), and brain (bottom) showing secretion and dispersion of mature melanosomes stained with GPNMB (green) into the stroma (S) surrounding the tumor (T), stained with HMB45 (red). Nuclei were stained blue with DAPI.

In primary melanoma, melanosome secretion was shown to promote the formation of the dermal metastatic niche [17]. However, the role of melanosomes in promoting metastasis of melanoma in later stages is mostly unknown. To further explore the pigmentation/melanosome function in melanoma progression, we tested clinical melanoma specimens. Since our unsupervised analysis that identified the four-melanoma subgroups was based on mRNA expression levels, we first confirmed the expression of melanogenesis genes at the protein level. Primary in situ melanoma tissues were immunostained for PMEL (SILV) using the HMB45 antibody. PMEL is a melanocyte-specific marker known to be a melanogenesis gene and is used in the pathological diagnosis of melanoma [19, 20]. PMEL is involved in the initiation of premelanosome production [21] and was also found in our analysis to be overexpressed in cluster 4 (Supplementary Table S4). PMEL strongly stained regions of melanoma (Fig. 3a), confirming its presence at the protein level. To further test whether the complete melanogenesis machinery is functional, indicated by the production of mature melanosomes, specimens were immunostained with mature melanosome marker, GPNMB [17]. Primary melanoma and the surrounding tissue clearly stained with GPNMB (Fig. 3a, left). This indicates that not only is the melanogenesis machinery active but also that melanosomes are actively secreted from melanoma into the stroma via a gradient pattern of diffusion from the epidermis (Fig. 3a, right).

Since our computational analysis showed that the machinery of melanin production in melanosomes highly correlated with poor prognosis, we further examined melanosome synthesis and function along a typical scheme of disease progression. In order to do this, we picked melanoma metastasis specimens in the lymph nodes, liver, and brain, from different patients. These tissues represent different stages of aggression [22]. Metastatic specimens were subjected to immunohistochemistry (IHC) for PMEL and GPNMB in order to follow melanosome production and distribution. Remarkably, metastases to the lymph, liver, and brain retained a hallmark pattern of melanosome secretion into the surrounding stroma (Fig. 3b). This indicates that melanosome production is retained throughout the progression of melanoma and that melanosomes are actively secreted to the tumor microenvironment. Taken together, our data demonstrate, for the first time, the presence of active production and secretion of melanosomes in distant metastatic sites, suggesting an important function for the melanosome organelle in the cancer metastases.

### A three-gene classifier for predicting melanoma molecular subtype

**Fig. 4:**
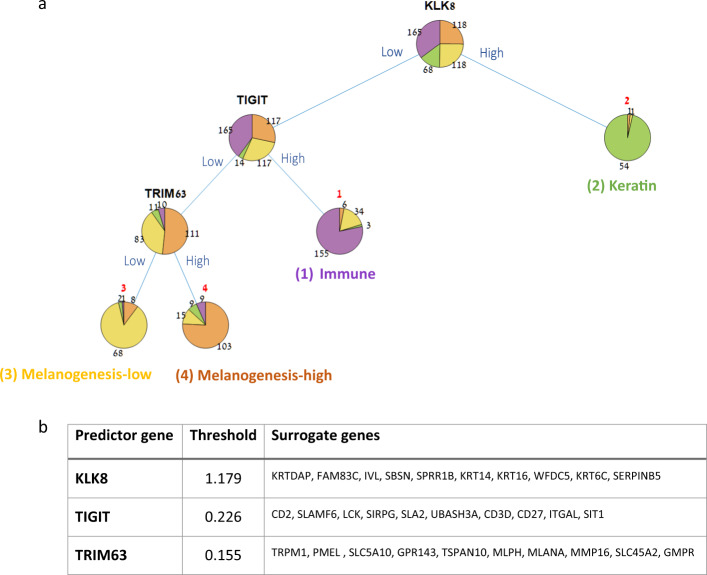
A three-gene decision tree for classifying melanoma samples. **a** The tree trained on the 469 TCGA samples. Classification of a new sample into one of the four subtypes is done by traversing the tree from its root to one of its leaves (representing an assignment to a subtype). Three biomarkers (shown in black) are used to determine the route along the tree: overexpression of KLK8 distinguishes the “Keratin” subtype, overexpression of TIGIT distinguishes the “Immune” subtype, and finally, overexpression of TRIM63 distinguishes the “Melanogenesis-high” from the “Melanogenesis-low” subtype. **b** Threshold values and surrogate genes for the three decision tree predictors as identified by the algorithm. Threshold values are used to distinguish between high and low values (based on normalized expression values), and surrogates can be used as alternatives for the respective predictor gene.

Having identified four distinct melanoma subgroups, each bearing a different survival risk and gene expression signature, we sought to develop a simple procedure to classify a new tumor into one of the four subgroups based on a minimal number of genes. We reasoned that such a procedure would be easier to interpret biologically than a 2000-gene signature and also cheaper to assay in diagnostics. We selected the decision tree classifier, which was often used in medical decision making due to its simplicity, easy interpretability, and robustness to outlier values [23]. In order to determine the number of genes to be used by our classifier, we trained a large number of decision trees on random subsets of the data and examined their performance as a function of the number of genes in the tree (Supplementary Fig. S5). Three genes gave a good tradeoff between classifier simplicity and performance. We then trained a three-gene decision tree on the full dataset, which achieved a training error of 0.187 (Fig. 4). Notably, the three genes selected by the tree-training algorithm, KLK8, TIGIT, and TRIM63, can be viewed as representatives of the three-gene expression signatures described earlier (Keratin, Immune, and Melanogenesis, respectively).

Remarkably, the genes identified as predictors by the decision tree have been previously associated with melanoma progression and prognosis: decrease in expression levels of kallikrein family member KLK8 was associated with the transfer from primary to metastatic melanoma [24], and its expression was linked to survival in various cancers [25–27]. TIGIT is a T-cell immunoreceptor with Ig and ITIM domains, which was recently identified as an attractive cancer immunotherapy target due to its central role in tumor immunosurveillance [28, 29]. Lastly, TRIM63 was implicated in melanoma cell migration/invasion [30]. Supplementary Fig. S6 provides a PCA visualization of the 469 melanoma samples projected to a three-dimensional space based on the expression levels of the three genes used in the decision tree. Supplementary Fig. S7 shows the RNA expression levels of the three genes on various normal tissues for reference, as obtained from The Human Protein Atlas [31, 32].

Interestingly, when we trained three-gene decision trees on 1000 random subsets obtained by resampling of the dataset samples, most trees had the same topology and contained predictors that are representatives of the three signatures (see Supplementary Figs. S8–S10).

### Experimental validation of predictor genes on patient cohort

The produced decision tree consists of three informative genes (KLK8, TIGIT, and TRIM63) along with a threshold level for each gene, which together provide a simple method for classifying melanoma tumors into one of the four subgroups. To classify a new tumor sample, one evaluates the sample’s expression levels for three predictor genes (biomarkers): first, a keratin predictor gene is evaluated (KLK8, or one of its keratinization surrogates such as KRT6C, IVL, SPRR1B, KRT14, KRT16), where high values would label the sample as “Keratin” and low values would lead to the next predictor. Next, an immune predictor is evaluated (TIGIT or one of its immune surrogates such as LCK, CD2, SLAMF6, SIRPG, SLA2, UBASH3A, CD3D, CD27, ITGAL, SIT1), where high values would label the sample as “Immune” and low levels would lead to the next and final predictor. Lastly, a melanogenesis predictor is evaluated (TRIM63 or one of its melanogenesis surrogates such as SLC45A2, PMEL, GPR143) where high values would label the sample as “Melanogenesis-high” and low values as “Melanogenesis-low.” We note that the distinction between low and high expression levels in each step of the classification process relies on gene-specific thresholds.

In order to validate the association between the classifier’s predictor genes and outcome, we experimentally tested their expression on six lymph node samples from patients of known outcomes. Patients who survived for 5 years or more after initial tumor diagnosis were defined as “good survival,” and those who survived 2 years or less after initial diagnosis as “poor survival” (Supplementary Table S5). In all clusters except for cluster 2 (the “Keratin” subgroup, which mostly corresponded to primary sites), lymph node tissues were identified in a substantial fraction of the samples (Supplementary Fig. S3b). For this reason, we tested the tree predictor genes in melanoma metastases to the lymph nodes from each patient using IHC.

**Fig. 5:**
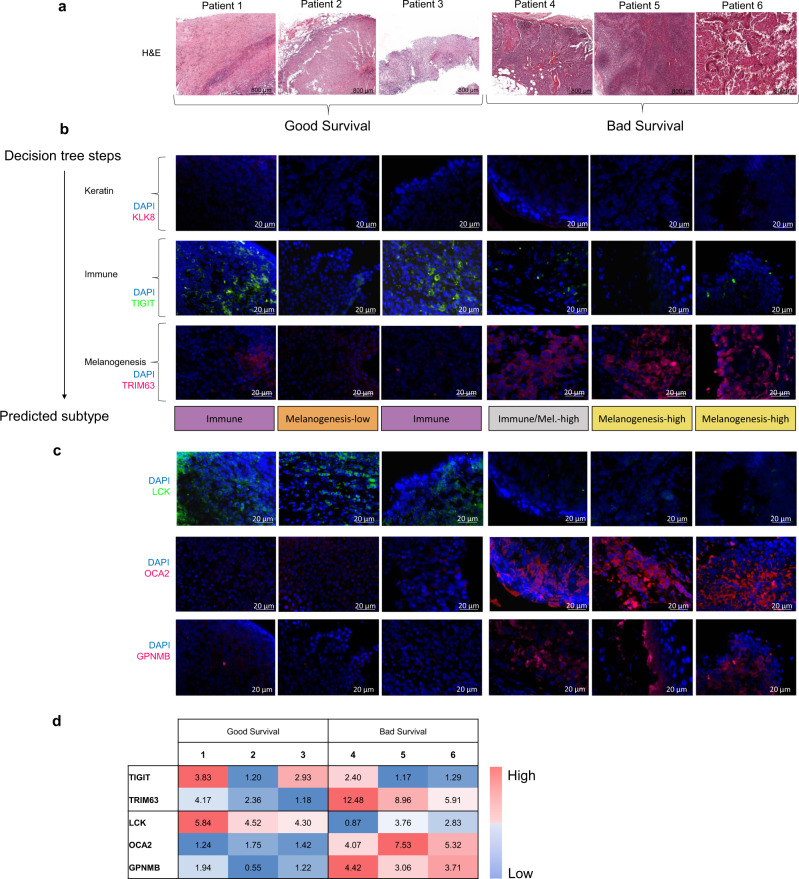
Melanogenesis and immune characteristics of melanoma metastases in good and poor prognostic outcomes. **a** Hematoxylin and eosin (H&E) staining of lymph nodes containing melanoma metastases from six different patients taken at 20× magnification. Patients 1–3 had good survival, while patients 4–6 survived poorly. **b** Immunohistochemical staining of the three proteins of the decision tree on the lymph node samples of the six patients. Nuclei were stained blue using DAPI. Row 1: using KLK8 (pink) as a predictor for the Keratin subgroup. Row 2: using TIGIT (Green) as a predictor of the Immune subgroup. Row 3: using TRIM63 (Pink) as a predictor of the Melanogenesis-high subgroup. The assignments of the specimens from the six patients to subtypes based on the expression levels of the three predictor genes are summarized as a label at the bottom bar. **c** Immunohistochemical staining of additional biomarkers for general prognosis. Row 1: LCK, an immune protein indicative of good prognostic outcome. Row 2: melanogenesis protein OCA2. Row 3: melanogenesis protein GPNMB. **d** Color matrix quantifying the fluorescence intensity of immunohistochemistry across biomarkers and patients. For each protein, values were independently normalized across the samples. (The KLK staining of all samples is negative, as they are all nonprimary, so KLK is not included here).

We first conducted hematoxylin and eosin (H&E) staining to confirm that the metastasis was, in fact, in the lymph nodes (Fig. 5a), and then stained each sample by the three predictor genes. Figure 5b shows the six tissue images per gene and Fig. 5d shows quantification of staining levels. All lymph node specimens stained negatively for the KLK8 gene, the predictor for the primary melanoma enriched “Keratin” subgroup in the tree (Fig. 5b, first row), indicating that the six samples do not belong to that subgroup. Staining for the TIGIT gene, the predictor for the “Immune” subgroup, appeared positive in the lymph node specimens of patients 1 and 3, thus assigning them to the best prognosis “Immune” subgroup based on the decision tree logic, in agreement with their good survival (Fig. 5b, second row). The specimens from patients 2, 5, and 6 stained negatively for TIGIT, excluding them from the “Immune” subgroup. The specimen from patient 4 showed borderline positive staining, making it difficult to classify. Finally, using TRIM63, the predictor for the “Melanogenesis-high” subgroup, specimens 5 and 6 were stained positively and were therefore assigned to the “Melanogenesis-high” subgroup, while specimen 3 that was stained negatively and therefore assigned to the “Melanogenesis-low” subgroup (Fig. 5b, third row). Except for patient 4, all patients were assigned to subgroups conferring relative survival in agreement with their known outcome. The results demonstrate the utility of biomarkers in prognostication of melanoma.

In addition to verifying the expression levels of proteins identified by the decision tree and their correlation to survival, we examined three other proteins that were identified as informative predictors for general prognosis (Fig. 5c). As an additional representative from the immune protein category we selected LCK, an Src family tyrosine kinase found on lymphocytes, that was previously identified as a biomarker for good prognosis in melanoma [12]. Indeed, patients with high LCK expression had a better prognosis. As additional representatives for the melanogenesis category, we selected GPNMB, indicative of mature melanosome presence [33], and OCA2, a transporter protein associated with melanocytes involved in melanin production and pH regulation of the melanosome [34]. Patients who had high levels of these proteins in their lymph nodes had worse outcomes associated with the “Melanogenesis-high” subgroup.

Finally, we repeated the experimental validation of the predictor genes on a second group of six melanoma samples (five metastatic and one primary) taken from patients with varying outcomes. As with the first sample group, we assigned each sample to a melanoma subgroup based on the decision tree logic and tested its association with patient outcome (Supplementary Fig. S11 and Supplementary Table S6). The protein expression levels of the examined predictor genes significantly varied among the various melanoma samples: KLK8’s expression levels enabled the assignment of the primary tumor sample to the keratin subgroup, while the expression levels of TIGIT and TRIM63 enabled the assignment of four out of the five remaining lymph node samples to subgroups conferring relative survival in agreement with their evaluated prognosis (Supplementary Fig. S11C).

Our data demonstrate that using the expression levels of only three classifier genes (keratin, immune, and melanogenesis) in our decision tree, we can reasonably predict the patient outcome using a lymph node biopsy. Our data further suggest the involvement of melanogenesis genes and the melanosome organelle in melanoma progression and lethality.

## Discussion

Our computational analysis of the 474 melanoma expression profiles identified four clinically distinct subgroups. The identified groups (Table 1) showed significant correspondence to TCGA’s transcriptomic classification [12]; however, TCGA’s keratin subgroup was split in our analysis into a keratin subgroup, composed mainly of primary tumors (cluster 2), and a melanogenesis-high subgroup, composed mainly of high-risk metastatic melanomas (cluster 4).

Three-gene expression signatures stratified the melanoma samples into the four clinically distinct subgroups: patients in cluster 1, characterized by high expression of immune genes, had the best survival, in agreement with previous reports in melanoma and other cancer types [12, 35].

Patients in cluster 2, characterized by a high expression of keratin related genes, had the worst survival. That cluster contained mostly primary samples. As noted by Akbani et al. [12], the poor survival can be attributed to the size bias of primary melanomas in the TCGA cohort.

The third expression pattern, which was of greatest interest to us, was enriched for melanogenesis and melanosome-related genes and distinguished the two metastasis-enriched clusters 3 (“Melanogenesis-low”) and 4 (“Melanogenesis-high”). Patients with high levels of the melanogenesis pattern were included in cluster 4 and had a worse survival rate compared to those in cluster 3, who had low levels. The association between overexpression of melanogenesis genes and poorer prognosis can be explained by several hypotheses: (1) trafficking of miRNA or other agents within secreted melanosomes by melanoma cells to their environment can make it more hospitable for melanoma progression [17]; (2) making the tumor resilient to chemotherapy, due to the drug-detoxifying properties of melanogenesis genes [18, 36]; or (3) removal of anticancer drugs from the melanoma cells by melanogenesis related transporters effluxing drugs outside of cells [37, 38]. The latter hypothesis is consistent with the fact that in our analysis, samples of the Melanogenesis-high cluster overexpressed ABC transporters such as ABCB5 and ABCC2 [37] (Supplementary Table S4). Our validation on samples from patients found that secretion of melanosomes to the surrounding tissues occurs both in primary melanoma (with clear gradient) as well as in metastatic melanoma. We therefore hypothesize that the reduced survival rate that characterizes the “Melanogenesis-high” subgroup is associated with the significantly higher activation of the melanogenesis pathway in these patients, as opposed to the “Melanogenesis-low” subgroup.

The importance of keratin, immune, and melanogenesis expression patterns in classifying melanoma tumors was also recognized in previous studies aimed at molecularly stratifying melanoma tumors. In 2010, Jönsson et al. identified four expression-based subgroups by analyzing 57 stage IV melanomas taken from patients [39]. These subgroups were called “normal-like,” “high-immune,” “pigmentation,” and “proliferative” sample subgroups. The normal-like group was characterized by overexpression of keratin genes (KRT17, KRT10, and KRT80), the high-immune group overexpressed immune genes (CCL13 and CD209), and the pigmentation group showed overexpression of melanogenesis genes (MITF, TYR, DCT, and MLANA). The proliferative group showed underexpression of the three signatures. The subgroups showed significant survival differences and were confirmed on additional patient cohorts [40–42]. A comparison of that classification to the one described here showed both commonalities and differences (see Supplementary Figs. S12–S15). Importantly, the keratin, immune, and melanogenesis expression patterns are manifest in both analyses, supporting their potential utility as biomarkers.

We trained a simple decision tree for classifying melanoma samples into one of the four subgroups. Our tests showed that a three-gene decision tree gave a good balance between classifier simplicity and accuracy. Although inferior in accuracy to more complex classifiers like SVM, a three-gene decision tree is easier to interpret biologically, easier to translate into a useful diagnostic kit in the future, and also captures the hierarchy of biological signals we identified in the data. A drawback for using a decision tree is that its thresholds depend on the distribution of the training data, and therefore must be recalculated before the tree can be applied to other datasets. Indeed, manual calibration of the decision thresholds was required to further validate the three-gene decision tree on the Lund melanoma dataset [41]. Once recalibrated, the decision tree successfully identified prognostic subgroups with corresponding biological characteristics on that second dataset (see Supplementary Figs. S16 and S17, and Supplementary Table S7).

Across multiple training runs, the trees produced tended to select one representative predictor gene from each of the three expression signatures. Key predictor genes, as well as their other signature representatives, were experimentally validated on a new cohort of melanoma taken from patients. Although limited in scope, the validation showed that the predictor genes differed in their protein expression levels among melanoma samples and confirmed the association of predictor levels with outcome. More substantial validation should be conducted on a larger cohort, composed of both primary and metastatic tumors, in order to validate the association of the identified expression signatures with outcome, to better evaluate the performance of the suggested classifier and to possibly identify better predictor genes.

We hope that classifiers such as the one suggested here will be translated in the near future into accurate and accessible diagnostic kits for improving the diagnosis and prognosis of melanoma tumors.

## Materials and methods

### Gene expression analysis for identification of melanoma subtypes

The expression profiles of 474 samples from TCGA’s melanoma RNA-Seq dataset [12] were downloaded from UCSC XENA’s web site in April 2018 (http://xena.ucsc.edu/), together with their associated clinical information (213 labels). We used the PROMO software suite (release 2019.5) [43, 44] for importing, preprocessing, analyzing, and visualizing the data. The downloaded RNA-Seq dataset (Illumina HiSeq platform, gene-level RSEM-normalized, log2 transformed) included 104 primary and 365 metastasis samples. Five samples were removed since they had inconsistent phenotype labels, and a variability-based filter was used to keep only the 2000 top variable genes. Clustering was performed on both samples and genes using the *k*-means algorithm with a correlation distance metric, using *k* = 4 for the samples and *k* = 5 for the genes. The algorithm was run 100 times and a solution minimizing the sum of point-to-centroid distances was chosen. The TCGA sample IDs included in each sample cluster are listed in Additional file 1.

We used PROMO’s multilabel analysis to evaluate the enrichment of the sample clusters for each of the clinical labels. Enrichment significance of sample clusters for categorical variables (such as sample type) was calculated using FDR-corrected [45] hypergeometric test. For numeric variables (such as age, Breslow’s depth, and pigmentation score), the difference between sample groups was evaluated using FDR-corrected Wilcoxon rank-sum test (Mann–Whitney *U* test). For exploring the prognostic value of the four sample clusters based on TCGA’s survival data, we used PROMO to plot 5-year survival curves using the Kaplan–Meier estimator [46], and calculated *p* values for the difference in survival for each group versus all other groups using the log-rank (Mantel–Haenszel) test [47, 48].

To identify active gene functions characterizing each of the sample clusters, we applied GO enrichment analysis [49] on the five gene clusters using both PROMO and the Expander software suite [50, 51]. To further characterize the biological function of the gene clusters, we also used Expander to test each gene cluster for enrichment for KEGG pathways [52].

Finally, to identify genes that were overexpressed on sample cluster 4 compared to all other samples, we applied the Wilcoxon rank-sum test on all dataset genes exhibiting nonzero variance (*n* = 20,227), and ranked all genes that were overexpressed on cluster 4 (in log scale) and showed *p* value < 1e−04 by decreasing fold change (difference between the mean expression in cluster 4 samples and all other samples). We used the GORILLA tool [53] for identifying the melanin biosynthesis genes appearing among the top 100 differentially expressed genes (Supplementary Table S4). Complete lists of overexpressed genes in each of the sample clusters appear in Additional file 2.

### Human histopathology and analysis of slides

Samples were obtained from patients at the E. Wolfson Medical Center and Tel Aviv Medical Center. The experimental study of the clinical samples was approved by the hospital ethics committees (from Wolfson Medical Center: approval number 0039-18WOMC; from Tel Aviv Medical Center: approval number 16-660-TLV6/7). Surgeons resected the primary tumors and the metastases and confirmed clear margins on the samples. Using demographic information, tumor characteristics, and length of survival following diagnosis, patients were identified as belonging to either good or bad survival groups by a pathologist. For the analysis of the first group of patients (Fig. 5), six patients were selected, and individual specimens from each patient were stained with either H&E or one of six different fluorescent antibodies (*n* = 6). For the second group (Supplementary Fig. S11), six additional patients were selected and specimens were also stained with either H&E or one of three different fluorescent antibodies (*n* = 6). Each picture presented in these two figures is from a different sample, and all samples in the same column are from the same patient. Specimens were fixed in formalin and subsequently embedded in paraffin. Hematoxylin (HHS16, Sigma-Aldrich) and eosin (HT110232, Sigma-Aldrich) staining of the samples was performed according to the manufacturer’s instructions. H&E images were obtained at 20× using Aperio Slide Scanner. Slides were first blocked and incubated with various combinations of primary antibodies including LCK (AF3704, R&D Systems), TIGIT (A700-047, Bethyl Laboratories), TRIM63 (bs2539R, Bioss), OCA2 (bs15510R, Bioss), GPNMB (AF2550, R&D Systems), HMB45 (ab732, Abcam), and KLK8 (MAB1719, R&D Systems). After subsequent washes, slides were incubated with the matching combinations of secondary antibodies, including Alexa Fluor 488 (A11055, Invitrogen), Alexa Fluor 594 (A21203, Invitrogen), and/or Alexa Fluor 647 (A31571, Invitrogen). 4′,6-diamidino-2-phenylindole (DAPI; Vector Laboratories) was then added dropwise to adequately visualize cell nuclei in the stained specimens. Images of slides were taken using fluorescence microscopy (Nikon) at 40× magnification, split into the individual color channels, and mean intensity of representative areas from each image was measured using ImageJ software. The mean intensity values recorded were then used to generate a color matrix demonstrating the level of expression of each protein in each patient’s sample.

For the analysis of melanosome spread and secretion, samples of human in situ melanoma, as well as metastases from different patients including brain, lymph, and liver were obtained from E. Wolfson Medical Center. Each image was generated from a separate patient at a different stage of the disease. Each metastasis location had one patient sampled for it (*n* = 1 per stage of disease). Immunohistochemical staining as described above was performed using GPNMB (AF2550, R&D Systems) and HMB45 (ab732, Abcam) as primary antibodies, and Alexa Fluor 488 (A11055, Invitrogen) and Alexa Fluor 594 (A21203, Invitrogen) as secondary antibodies, with DAPI (Vector Laboratories) added at the end. Images of the slides were taken at 20× magnification using a Nikon fluorescent microscope. The image of in situ melanoma was then broken into its component color channels using ImageJ software, and four equally sized, equidistant frames were cut out and measured for the mean intensity of GPNMB to quantify the gradient of its diffusion from the primary tumor.

### Training of a gene expression-based decision tree classifier

To train a molecular classifier for predicting melanoma subgroups, we used the expression levels of the 2000 most variable genes on the set of 469 melanoma samples. We used Matlab’s implementation (R2019a) (accessed through PROMO [54]) to grow a classification tree using a curvature test as the method for splitting predictors [55, 56]. The training procedure consisted of two steps. First, we assessed the best number of predictor genes to be included in the decision tree, by training many trees on randomly selected subsets of the dataset samples (90% of the samples were included in each iteration) while varying the number of allowed predictor genes and the pruning level. The average training error was calculated for each tree size. Having determined the number of predictor genes, we then used the entire dataset samples (*n* = 469) to train the final decision tree. To evaluate the robustness of the decision tree, we repeated the procedure multiple times and compared the resulting tree configurations and the selected predictor genes and their biological categories.

## Supplementary information

Supplementary Information

The assignments of samples and genes to clusters, as they appear on Figure 1.

Lists differentially expressed genes for each melanoma subgroup (as appearing in Figure 1) versus all other subgroups
