# Supplementary material for: Classification of node-positive melanomas into prognostic subgroups using keratin, immune, and melanogenesis expression patterns
Source: Oncogene. 2021 Feb 9;40(10):1792–805. doi: 10.1038/s41388-021-01665-0 (PMC7946641; doi:10.1038/s41388-021-01665-0)
Supplement: Supplementary file 1 — Supplementary Information [file 41388_2021_1665_MOESM1_ESM.pdf]

Supplementary Information

Classification of node-positive melanomas into prognostic subgroups using keratin, immune and melanogenesis expression patterns

Dvir Netanely[1], Stav Leibou[2], Roma Parikh[2], Neta Stern[1], Hananya Vaknine[3], Ronen Brenner[3], Sarah Amar[3], Rivi Haiat Factor[4], Tomer Perluk[4], Jacob Frand[4], Eran Nizri[5,2], Dov Hershkovitz[6,2], Valentina Zemser-Werner[6], Carmit Levy[2], Ron Shamir[1*]

[1]Blavatnik School of Computer Science, Tel Aviv University, Tel Aviv, Israel

[2]Sackler Faculty of Medicine, Tel Aviv University, Tel Aviv, Israel

[3]Department of Oncology, Edith Wolfson Medical Center, Holon, Israel

[4]Department of Plastic and Reconstructive Surgery, Edith Wolfson Medical Center, Holon, Israel

[5]Department of Surgery A, Tel Aviv Sourasky Medical Center, Tel Aviv, Israel

[6]Institute of Pathology, Tel Aviv Sourasky Medical Center, Tel Aviv, Israel

*Corresponding author, e-mail: rshamir@tau.ac.il

Supplementary Information - Section 1

Supplementary Figures and Tables

		Cluster 1	Cluster 2	Cluster 3	Cluster 4	Total
Sample number		**105**	**68**	**118**	**118**	**469**
Tissue site	**Primary Tumor**	16	53	10	25	**104**
	**Regional Cutaneous or Subcutaneous**	21	6	32	15	**74**
	**Regional Lymph Node**	115	2	57	49	**223**
	**Distant Metastasis**	13	7	19	29	**68**
TCGA's Transcriptomic subtypes	**Immune**	114	2	34	18	**168**
	**Keratin**	1	41	2	56	**100**
	**MITF-Low**	0	0	57	2	**59**
	**NA**	50	23	25	41	**139**

Table S1: Characterization of the four melanoma subgroups.

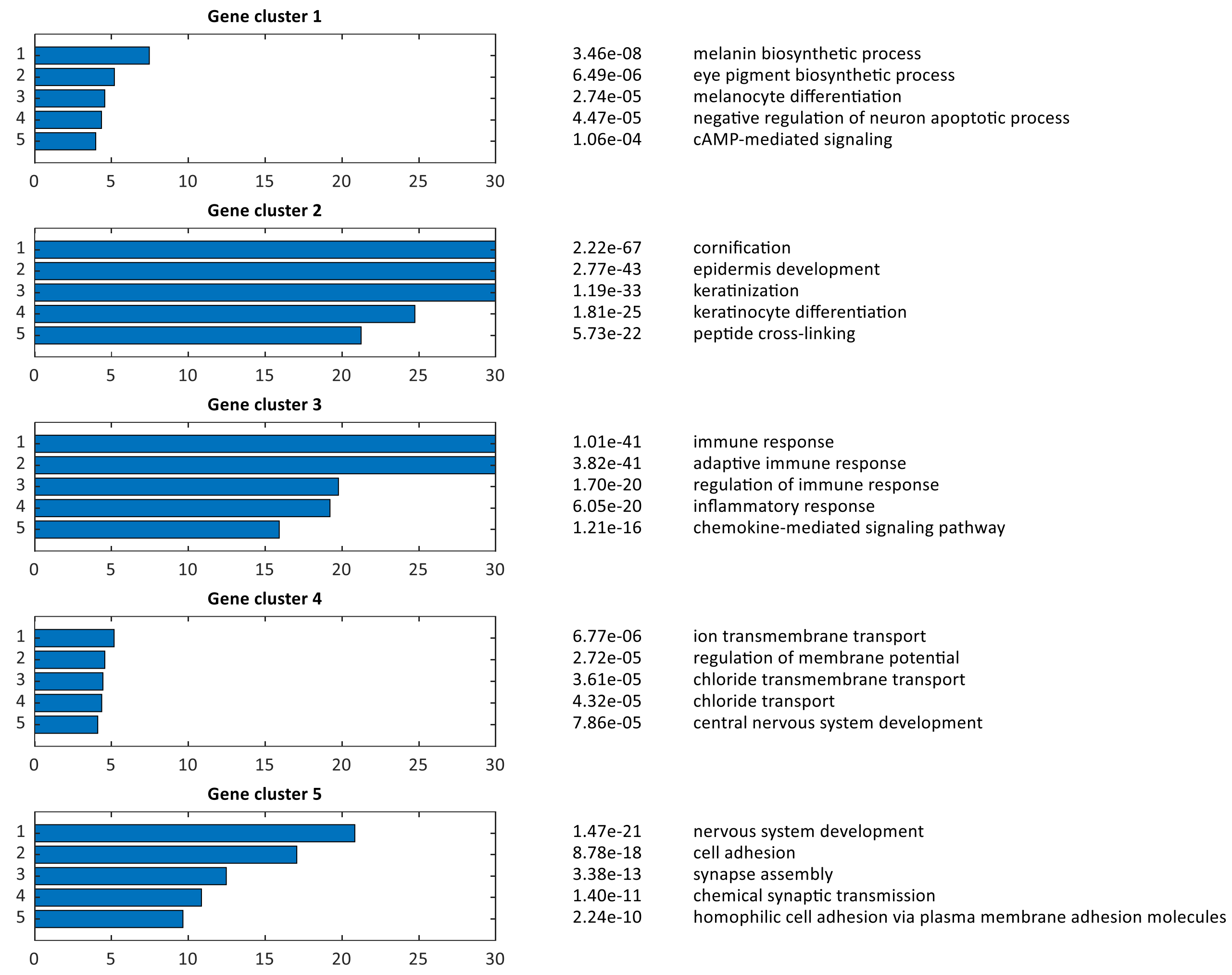

Figure S1: Gene Ontology enrichments on the five gene clusters. The analysis was performed in PROMO[1]. The five most significant GO terms, along with their FDR-corrected hypergeometric test p-values are listed for each gene cluster. Gene clusters 1, 2, and 3 were significantly enriched for melanogenesis, keratinization, and immune GO terms, respectively.

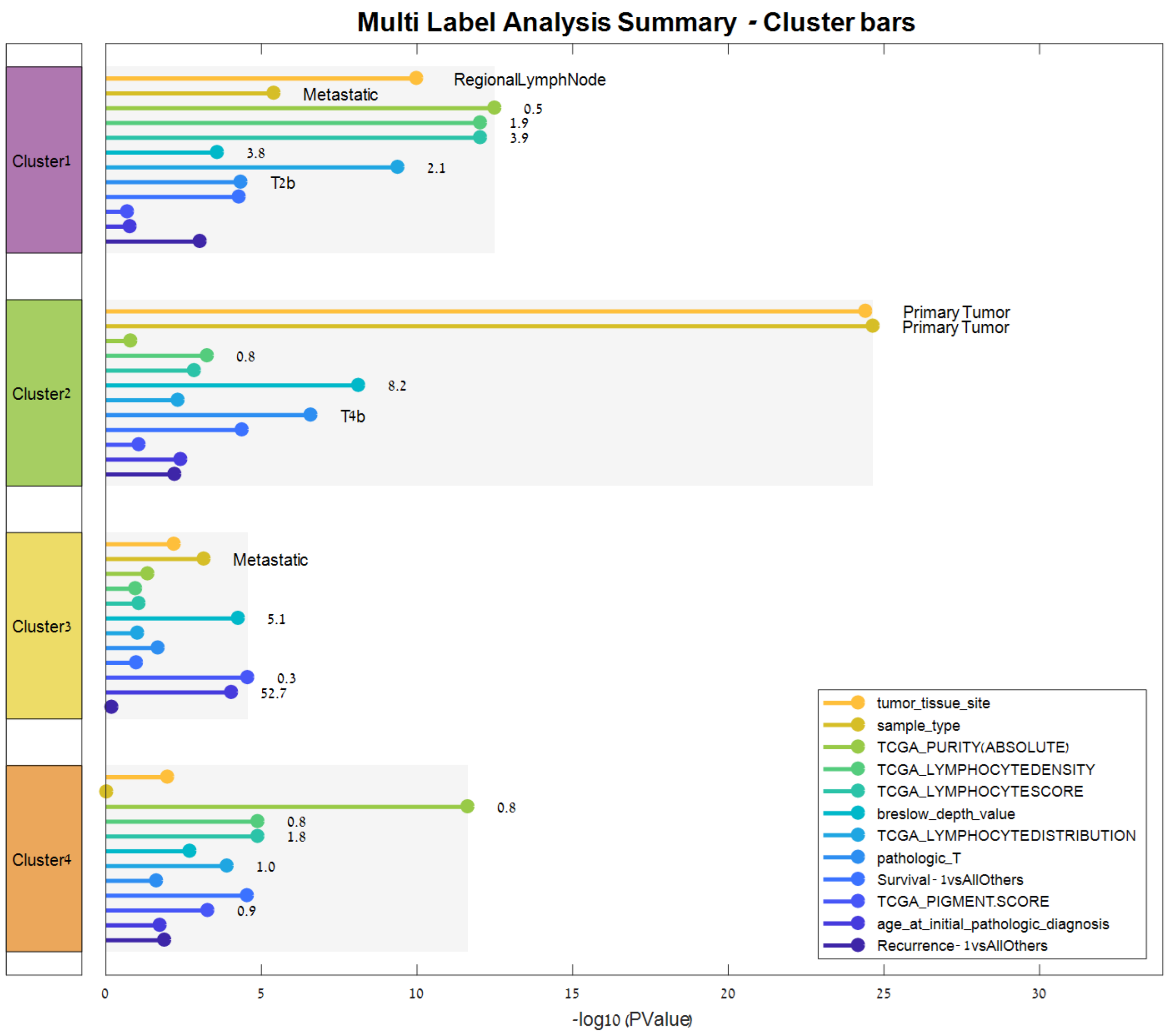

Figure S2: Enrichment for clinical labels on the four melanoma sample clusters. The analysis was performed using PROMO's multi-label sample-cluster analysis[1], which scanned all the clinical labels available for the samples and identified significant label enrichments for each sample cluster. P-values are significance of enrichments after FDR correction. Sample cluster 2 was significantly enriched for primary tumors.

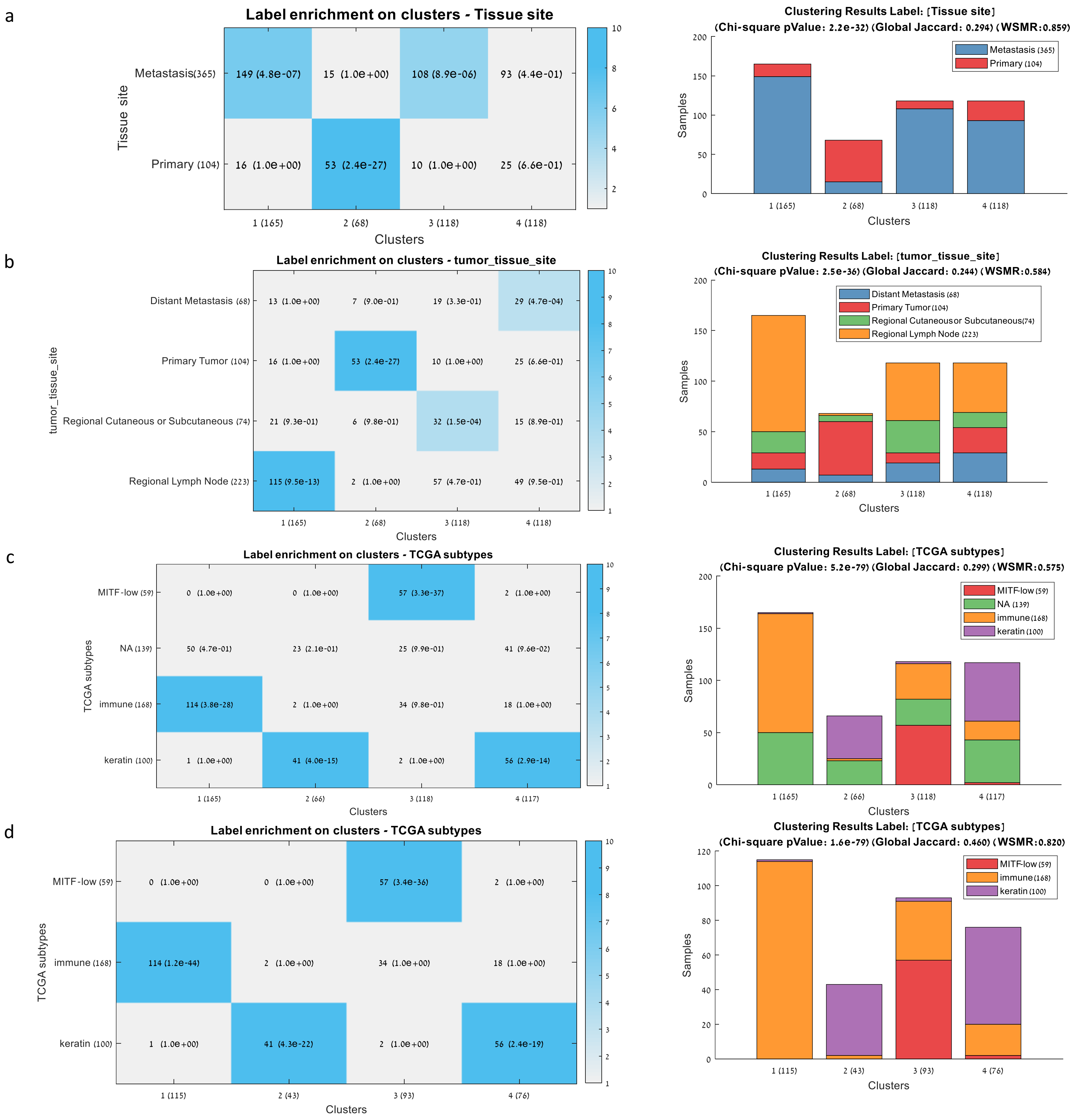

Figure S3: Characteristics of the four melanoma subtypes. Concordance between the four melanoma subgroups, tumor tissue sites, and TCGA's three transcriptomic subgroup labels. For each comparison, the histogram on the right shows the breakdown of samples in each subtype into categories, and the matrix on

the left shows the confusion matrix. For each cell, the number of samples and FDR-corrected p-value for enrichment based on the hypergeometric test is shown. Color scale is –log10(p-value). **(a)** Primary vs. Metastasis **(b)** Detailed tissue site **(c)** TCGA's three transcriptomic subtypes, including NA value for new samples that were not included in TCGA's melanoma paper[2] **(d)** TCGA's three transcriptomic subtypes, omitting the NA samples.

Gene Cluster	GO Term	#genes	Raw p-value	Empirical p-Value	Gene List
1	neurogenesis - GO:0022008	68	7.10E-14	2.00E-04	[GABRB3, ROBO2, ONECUT2, LAMC3, CTNND2, RASGRF1, PCSK9, KNDC1, NR2E1, DUSP15, CDH4, SOX1, DPYSL4, CDH1, CRTAC1, SALL4, EFHD1, NRTN, PLXNC1, PITX2, SOX6, SH3GL2, MNX1, EPHA5, NKX2-8, SERPINF1, ADRA2C, MAPK8IP2, POU3F1, ISL1, OLFM1, NRG3, DCT, RTN4RL1, KIT, LMX1B, MAPT, HAP1, BRSK2, ARX, PRDM13, PRELP, ADCY1, FSTL4, DLL3, TRPM1, VTN, GBX2, CTNNA2, PROM1, WNT4, SPTBN2, NKX2-2, MCOLN3, SYT3, GABRA5, MCF2, FZD9, DCDC2, L1CAM, POU4F1, GNAO1, CPEB1, NR4A3, NEURL1, RAB17, MDGA2, BMPR1B]
	somatodendritic compartment - GO:0036477	34	1.60E-08	4.00E-04	[PCSK2, CHRM1, CTTNBP2, CTNND2, CACNA1B, ADCY2, KNDC1, CPN1, KCNN1, KCNN2, PPARGC1A, ANKS1B, KCNH1, SPTBN2, SYT5, EPHA5, MME, GABRA5, SERPINF1, MAPK8IP2, L1CAM, GNAO1, OLFM1, CPEB1, MLPH, NOV, RTN4RL1, NEURL1, EEF1A2, RAB17, MAPT, BMPR1B, CRYAB, PDE9A]
	melanosome membrane - GO:0033162	6	2.34E-08	4.00E-04	[OCA2, SLC45A2, GPR143, DCT, TYRP1, TYR]
	cell body - GO:0044297	28	2.70E-08	6.00E-04	[PCSK2, CTNND2, CACNA1B, KNDC1, CPN1, KCNN1, KCNN2, PPARGC1A, KCNH1, SPTBN2, SYT5, EPHA5, GABRA5, SERPINF1, MAPK8IP2, L1CAM, GNAO1, OLFM1, CPEB1, NOV, RTN4RL1, NEURL1, EEF1A2, RAB17, MAPT, BMPR1B, CRYAB, PDE9A]
	secondary metabolic process - GO:0019748	9	4.43E-08	6.00E-04	[OCA2, SLC45A2, AS3MT, ABCC2, CITED1, DCT, TYRP1, CYP1A1, TYR]
	anatomical structure morphogenesis - GO:0009653	66	4.48E-08	6.00E-04	[ROBO2, RYR1, ONECUT2, CITED1, LAMC3, CTNND2, ONECUT1, LDB3, KNDC1, NR2E1, MYLK3, CDH4, SOX1, DPYSL4, KRT27, SALL4, RBPMS2, CAPN3, NRTN, PITX2, SOX6, SH3GL2, MNX1, KCNH1, EPHA5, NKX2-8, RIPK4, MAPK8IP2, MMP8, ISL1, OLFM1, CEACAM1, NOV, NRG3, SFRP5, ITGA7, CRYAB, MET, BRSK2, SLC24A4, HPGD, ARX, KLK4, GATA4, PRELP, ADCY1, CACNA1H, DLL3, TRPM1, VTN, GBX2, MYH14, TNNI3, CTNNA2, PROM1, WNT4, SPTBN2, CAP2, FOXF2, L1CAM, POU4F1, NR4A3, NOX5, RADIL, BMPR1B, RAMP1]
	neuron projection development - GO:0031175	31	4.80E-08	6.00E-04	[ROBO2, BRSK2, CTNND2, ARX, RASGRF1, PRELP, NR2E1, ADCY1, CDH4, DPYSL4, GBX2, CDH1, CRTAC1, EFHD1, NRTN, CTNNA2, SH3GL2, MNX1, SPTBN2, EPHA5, NKX2-8, MCF2, MAPK8IP2, L1CAM, POU4F1, ISL1, GNAO1, NR4A3, RTN4RL1, MAPT, BMPR1B]
	central nervous system development - GO:0007417	39	4.91E-08	6.00E-04	[ROBO2, CITED1, LAMC3, CTTNBP2, ARX, KNDC1, NR2E1, VTN, SOX1, GBX2, CDH1, CRTAC1, CTNNA2, SOX6, PITX2, PPARGC1A, SH3GL2, MNX1, NKX2-2, WNT4, SPTBN2, EPHA5, SLC6A17, S100A1, GABRA5, POU3F1, POU4F1, ISL1, GNAO1, BCAN, NR4A3, NRG3, DCT, RTN4RL1, NEURL1, MAL, HAP1, MAPT, MDGA2]
	melanin metabolic process - GO:0006582	6	7.19E-08	8.00E-04	[OCA2, SLC45A2, CITED1, DCT, TYRP1, TYR]
	developmental pigmentation - GO:0048066	8	2.97E-07	0.0034	[OCA2, SLC45A2, GPR143, CITED1, DCT, KIT, TYRP1, TYR]
2	skin development - GO:0043588	85	4.46E-72	2.00E-04	[FOXE1, ITGB4, KRT23, ABCA12, TGM1, LCE1B, CASP14, PRSS8, TGM5, KRT6C, TGM3, RPTN, TP63, KRT6B, KRT6A, DSP, KRT4, KRT2, KRT1, SPINK5, KRT79, KRT78, KRT7, KRT77, KRT5, OVOL1, KRT75, LOR, LCE1C, EREG, LCE2B, FLG2, LCE2C, CLDN4, LCE2A, DSG1, PKP1, PKP3, DSG3, IRF6, DSC1, IVL, DSC2, S100A7, DSC3, SPRR2E, FLG, SPRR3, SPRR2G, CSTA, KRT80, KLK5, ALOX12B, APCDD1, EVPL, KLK8, PPL, EGFR, LCE3D, SCEL, PERP, SFN, PI3, SPRR2A, SPRR2B, ALOXE3, SPRR2D, CDSN, C1orf68, KLK13, KRT13, GRHL3, KRT10, CNFN, ASPRV1, LCE3E, KRT19, KRT17, GJB3, KRT16, KRT15, KRT14, SPRR1A, FGFR2, SPRR1B]
	epidermal cell differentiation - GO:0009913	72	6.99E-62	2.00E-04	[KRT23, ABCA12, TGM1, LCE1B, CASP14, PRSS8, TGM5, KRT6C, TGM3, RPTN, TP63, KRT6B, KRT6A, DSP, KRT4, KRT2, KRT1, SPINK5, KRT79, KRT78, KRT7, KRT77, KRT5, KRT75, LOR, LCE1C, EREG, LCE2B, LCE2C, LCE2A, DSG1, PKP1, PKP3, DSG3, IRF6, DSC1, IVL, DSC2, S100A7, DSC3, SPRR2E, FLG, SPRR3, SPRR2G, CSTA, KRT80, KLK5, EVPL, KLK8, PPL, LCE3D, SCEL, PERP, SFN, PI3, SPRR2A, SPRR2B, SPRR2D, CDSN, C1orf68, KLK13, KRT13, KRT10, CNFN, LCE3E, KRT19, KRT17, KRT16, KRT15, KRT14, SPRR1A, SPRR1B]
	cornified envelope - GO:0001533	40	1.51E-55	2.00E-04	[SPRR2E, FLG, SPRR3, CSTA, SPRR2G, EVPL, CST6, PPL, TGM1, SCEL, LCE3D, LCE1B, PI3, SPRR2A, SPRR2B, RPTN, SPRR2D, DSP, CDSN, KRT2, C1orf68, KRT1, KRT10, CNFN, LOR, LCE3E, LCE1C, LCE2B, LCE2C, LCE2A, PKP1, DSG1, DSG3, PKP3, SPRR1A, DSC1, IVL, SPRR1B, DSC2, DSC3]
	epithelial cell differentiation - GO:0030855	87	3.37E-53	2.00E-04	[EHF, KRT23, TFCP2L1, ABCA12, TGM1, LCE1B, CASP14, PRSS8, TGM5, KRT6C, TGM3, RPTN, TP63, KRT6B, KRT6A, DSP, KRT4, KRT2, KRT1, SPINK5, KRT79, KRT78, KRT7, KRT77, FOXL2, KRT5, KRT75, LOR, LCE1C, EREG, LCE2B, LCE2C, LCE2A, ELF3, DSG1, PKP1, PKP3, DSG3, IRF6, RHCG, DSC1, IVL, DSC2, S100A7, DSC3, SPRR2E, FLG, SPRR3, SPRR2G, CSTA, KRT80, DLX3, KLK5, EVPL, KLK8, PPL, LCE3D, SCEL, RAB25, PERP, SFN, PI3, SPRR2A, SPRR2B, SPRR2D, CDSN, PSAPL1, WNT7B, AKR1C1, C1orf68, KLK13, KRT13, AKR1C2, PTK6, KRT10, CNFN, GRHL2, LCE3E, KRT19, KRT17, KRT16, KRT15, KRT14, CD24, SPRR1A, FGFR2, SPRR1B]
	peptide cross-linking - GO:0018149	28	3.95E-34	2.00E-04	[SPRR2E, FLG, SPRR3, CSTA, EVPL, TGM1, LCE3D, LCE1B, PI3, SPRR2A, TGM5, SPRR2B, TGM3, SPRR2D, DSP, KRT2, C1orf68, KRT1, KRT10, LOR, LCE3E, LCE1C, LCE2B, LCE2C, LCE2A, SPRR1A, IVL, SPRR1B]
	extracellular exosome - GO:0070062	118	5.88E-23	2.00E-04	[CALML5, CBLC, CALML3, DEFB1, AQP5, TGM1, CKMT1B, PRSS8, PRSS3, KRT6C, TGM3, KRT6B, CD177, KRT6A, GBP6, ENTPD2, KRT2, KRT1, SPINK5, SLC6A14, KRT79, KRT78, KRT7, KRT77, KRT5, NCCRP1, KRT75, SULT2B1, FLG2, SLPI, SPINT1, SCNN1B, SCNN1A, PKP1, RHCG, S100A9, DSC1, S100A8, DSC2, S100A7, KPRP, CRABP2, TACSTD2, SLC5A1, EVPL, PPL, FUT3, PKHD1, SFN, PI3, S100A14, CLCA4, PROM2, GGT6, CDSN, C1orf68, A2ML1, CNFN, CRNN, AKR1B10, LCN2, FAT2, SPRR1B, LAD1, CLIC3, PCDHGB5, ITGB4, DMKN, CASP14, CTSG, ITGB6, SERPINB3, DSP, SERPINB4, LYNX1, GPX2, MMP7, ARG1, ANXA3, SERPINB5, TMPRSS11D, EPN3, CEACAM5, DSG1, IRF6, DSG3, IVL, SPRR3, LRRC15, CSTA, SERPINB13, SBSN, CST6, SCEL, RAB25, RNASE7, MAL2, EPS8L2, LGALS7B, WNT7B, AKR1C1, CKMT1A, KLK13, KRT13, BBOX1, KRT10, PDZK1IP1, SLURP1, KLK11, KRT19, KRT17, KRT16, KRT15, KRT14, SAA1, S100P, NECTIN4, C1orf116]
	extracellular vesicle - GO:1903561	118	9.25E-23	2.00E-04	[CALML5, CBLC, CALML3, DEFB1, AQP5, TGM1, CKMT1B, PRSS8, PRSS3, KRT6C, TGM3, KRT6B, CD177, KRT6A, GBP6, ENTPD2, KRT2, KRT1, SPINK5, SLC6A14, KRT79, KRT78, KRT7, KRT77, KRT5, NCCRP1, KRT75, SULT2B1, FLG2, SLPI, SPINT1, SCNN1B, SCNN1A, PKP1, RHCG, S100A9, DSC1, S100A8, DSC2, S100A7, KPRP, CRABP2, TACSTD2, SLC5A1, EVPL, PPL, FUT3, PKHD1, SFN, PI3, S100A14, CLCA4, PROM2, GGT6, CDSN, C1orf68, A2ML1, CNFN, CRNN, AKR1B10, LCN2, FAT2, SPRR1B, LAD1, CLIC3, PCDHGB5, ITGB4, DMKN, CASP14, CTSG, ITGB6, SERPINB3, DSP, SERPINB4, LYNX1, GPX2, MMP7, ARG1, ANXA3, SERPINB5, TMPRSS11D, EPN3, CEACAM5, DSG1, IRF6, DSG3, IVL, SPRR3, LRRC15, CSTA, SERPINB13, SBSN, CST6, SCEL, RAB25, RNASE7, MAL2, EPS8L2, LGALS7B, WNT7B, AKR1C1, CKMT1A, KLK13, KRT13, BBOX1, KRT10, PDZK1IP1, SLURP1, KLK11, KRT19, KRT17, KRT16, KRT15, KRT14, SAA1, S100P, NECTIN4, C1orf116]
	extracellular organelle - GO:0043230	118	1.00E-22	2.00E-04	[CALML5, CBLC, CALML3, DEFB1, AQP5, TGM1, CKMT1B, PRSS8, PRSS3, KRT6C, TGM3, KRT6B, CD177, KRT6A, GBP6, ENTPD2, KRT2, KRT1, SPINK5, SLC6A14, KRT79, KRT78, KRT7, KRT77, KRT5, NCCRP1, KRT75, SULT2B1, FLG2, SLPI, SPINT1, SCNN1B, SCNN1A, PKP1, RHCG, S100A9, DSC1, S100A8, DSC2, S100A7, KPRP, CRABP2, TACSTD2, SLC5A1, EVPL, PPL, FUT3, PKHD1, SFN, PI3, S100A14, CLCA4, PROM2, GGT6, CDSN, C1orf68, A2ML1, CNFN, CRNN, AKR1B10, LCN2, FAT2, SPRR1B, LAD1, CLIC3, PCDHGB5, ITGB4, DMKN, CASP14, CTSG, ITGB6, SERPINB3, DSP, SERPINB4, LYNX1, GPX2, MMP7, ARG1, ANXA3, SERPINB5, TMPRSS11D, EPN3, CEACAM5, DSG1, IRF6, DSG3, IVL, SPRR3, LRRC15, CSTA, SERPINB13, SBSN, CST6, SCEL, RAB25, RNASE7, MAL2, EPS8L2, LGALS7B, WNT7B, AKR1C1, CKMT1A, KLK13, KRT13, BBOX1, KRT10, PDZK1IP1, SLURP1, KLK11, KRT19, KRT17, KRT16, KRT15, KRT14, SAA1, S100P, NECTIN4, C1orf116]
	structural molecule activity - GO:0005198	54	5.09E-21	2.00E-04	[LAD1, WWC1, KRT23, LCE1B, EPB41L4B, KRT6C, KRT6B, KRT6A, DSP, KRT4, KRT2, KRT1, KRT79, KRT78, KRT7, KRT77, KRT5, KRT75, LOR, LCE1C, LCE2B, FLG2, LCE2C, CLDN4, LCE2A, PKP1, INA, IVL, SHANK2, SPRR2E, FLG, SPRR3, CSTA, KRT80, EVPL, PPL, LCE3D, MAL2, PI3, SPRR2A, SPRR2B, SPRR2D, C1orf68, KRT13, KRT10, LCE3E, MPP7, KRT19, KRT17, KRT16, KRT15, KRT14, SPRR1A, SPRR1B]
	intermediate filament - GO:0005882	28	4.63E-18	2.00E-04	[FLG, KRT80, KRT23, CASP14, KRT6C, KRT6B, KRT6A, DSP, KRT4, KRT2, KRT1, KRT13, KRT79, KRT78, KRT7, KRT77, KRT10, KRT5, KRT75, KRT19, KRT17, KRT16, KRT15, KRT14, PKP1, EPPK1, INA, SHANK2]
3	immune system process - GO:0002376	176	8.58E-85	2.00E-04	[FCN1, ADAMDEC1, FCMR, NCF1, ATP8A1, CLEC10A, AQP9, SIRPG, HP, LY75, PRF1, RORC, SLA2, CXCL13, IKZF3, CLU, IFI44L, VPREB3, GPR174, CYSLTR2, SIT1, RGS1, TBC1D10C, TNFSF11, HLA-DOA, HLA-DOB, ZNF683, ZBP1, GBP5, CD96, LAG3, PRKCB, THEMIS, HLA-G, LAX1, CHIT1, FCAMR, CD8B, IFI27, CD8A, PADI2, PRKCQ, CLEC4E, CARD11, SKAP1, IDO1, BLK, TNFRSF11B, GATA3, CD1C, LY9, PLAC8, SPTA1, C3, CD79B, CD79A, KLRK1, C7, UBD, CD19, BTLA, NLRP2, SLAMF7, TNFRSF17, SLAMF6, ICOS, HLA-DQA2, HLA-DQA1, SLAMF1, HLA-DRB5, SIGLEC14, KLRC2, BCL11B, TNFRSF9, CRTAM, SH2D1A, IFNLR1, LYZ, SELE, SELP, MARCO, CXCL10, CXCL11, PTPRC, SELL, CD27, KLRD1, IL7R, HAMP, PIGR, ITK, CIITA, CXCL9, TNFRSF13B, FASLG, CD3G, CTSW, PTPN22, LRMP, CD3E, ITGAL, CD3D, PIK3CG, JCHAIN, TNFSF13B, SPN, GNLY, KYNU, OLR1, CTLA4, CD38, CCR7, LBP, CCR5, CCR2, CR2, CR1, ITGA4, RHOH, PAX5, MMP9, ZAP70, HSH2D, AIM2, ITGAD, IFNG, LCK, BANK1, IL1B, XCL2, CHI3L1, TLR8, CD48, TLR10, LTB, SMPDL3B, MS4A1, HLA-DQB2, LTF, BIRC3, CCL14, CD5L, FGL2, CXCR5, CST7, IL2RG, LILRA3, LILRA4, CCL8, CCL5, CXCR3, TBX21, IL21R, CCL19, CCL18, IL12RB1, GBP1, IL33, PLA2G2D, CCL22, CCL21, ERAP2, TRAT1, CD70, GZMA, GZMB, LILRB1, GZMH, CD2, CD6, CD5, CAMK4, POU2AF1, CD7, CD247, PDCD1]
	regulation of immune system process - GO:0002682	116	3.02E-57	2.00E-04	[FCN1, CLEC10A, SIRPG, SLA2, CXCL13, IKZF3, CLU, SIT1, GPR171, TBC1D10C, TNFSF11, UBASH3A, HLA-DOA, HLA-DOB, ZNF683, GBP5, CD96, LAG3, PRKCB, THEMIS, HLA-G, LAX1, CD8B, CD8A, PADI2, PRKCQ, CLEC4E, CARD11, SKAP1, IDO1, BLK, KLRB1, GATA3, CD1C, KIR2DL4, SPTA1, C3, CD79B, CD79A, KLRK1, C7, CD19, BTLA, SLAMF7, STAP1, SLAMF6, ICOS, HLA-DQA2, HLA-DQA1, SLAMF1, HLA-DRB5, CRTAM, SH2D1A, IFNLR1, SELP, MARCO, CXCL10, CXCL11, PTPRC, SELL, CD27, KLRD1, IL7R, PIGR, ITK, CXCL9, TNFRSF13B, CD3G, PTPN22, CD3E, ITGAL, CD3D, TNFSF13B, SPN, CTLA4, CD38, CCR7, LBP, CCR2, CR2, CR1, ITGA4, MMP12, GREM1, FCER2, ZAP70, AIM2, IFNG, LCK, BANK1, IL1B, TLR8, CD48, TLR10, SMPDL3B, HLA-DQB2, LTF, BIRC3, CCL5, CXCR3, TBX21, CCL19, TIGIT, IL12RB1, GBP1, IL33, PLA2G2D, CCL21, TRAT1, LILRB1, CD2, CD6, CD5, CAMK4, CD247, PDCD1]
	regulation of cell activation - GO:0050865	61	1.40E-37	2.00E-04	[TNFRSF13B, SIRPG, PTPN22, CD3G, CD3E, SLA2, CD3D, IKZF3, TNFSF13B, SPN, SIT1, TBC1D10C, CD38, TNFSF11, CTLA4, CCR7, LBP, HLA-DOA, CCR2, ZNF683, LAG3, HLA-G, LAX1, ZAP70, IFNG, BANK1, LCK, IL1B, PRKCQ, HLA-DQB2, CARD11, IDO1, GATA3, SPTA1, KLRK1, CCL5, TBX21, BTLA, STAP1, CCL19, TIGIT, IL12RB1, ICOS, HLA-DQA2, HLA-DQA1, SLAMF1, IL33, PLA2G2D, HLA-DRB5, CCL21, LILRB1, CD2, SELP, PTPRC, CD6, CD5, CAMK4, CD27, CD247, PDCD1, IL7R]
	regulation of leukocyte cell-cell adhesion - GO:1903037	51	1.03E-36	2.00E-04	[SIRPG, PTPN22, CD3G, CD3E, CD3D, TNFSF13B, SPN, SIT1, TNFSF11, CTLA4, CCR7, HLA-DOA, CCR2, ZNF683, LAG3, ITGA4, HLA-G, LAX1, ZAP70, IFNG, LCK, IL1B, PRKCQ, HLA-DQB2, CARD11, IDO1, GATA3, SPTA1, KLRK1, CCL5, BTLA, CCL19, TIGIT, IL12RB1, ICOS, HLA-DQA2, HLA-DQA1, SLAMF1, PLA2G2D, HLA-DRB5, CCL21, LILRB1, CD2, PTPRC, CD6, CD5, CAMK4, CD27, CD247, PDCD1, IL7R]
	regulation of T cell activation - GO:0050863	50	1.36E-36	2.00E-04	[SIRPG, PTPN22, CD3G, CD3E, CD3D, TNFSF13B, SPN, SIT1, TNFSF11, CTLA4, CCR7, HLA-DOA, CCR2, ZNF683, LAG3, HLA-G, LAX1, ZAP70, IFNG, LCK, IL1B, PRKCQ, HLA-DQB2, CARD11, IDO1, GATA3, SPTA1, KLRK1, CCL5, BTLA, CCL19, TIGIT, IL12RB1, ICOS, HLA-DQA2, HLA-DQA1, SLAMF1, PLA2G2D, HLA-DRB5, CCL21, LILRB1, CD2, PTPRC, CD6, CD5, CAMK4, CD27, CD247, PDCD1, IL7R]
	positive regulation of leukocyte activation - GO:0002696	45	8.39E-32	2.00E-04	[SIRPG, CD3G, GATA3, CD3E, CD3D, TNFSF13B, SPN, SPTA1, KLRK1, CCL5, TBX21, BTLA, CD38, TNFSF11, CTLA4, STAP1, CCR7, CCL19, LBP, IL12RB1, ICOS, HLA-DQA2, HLA-DQA1, SLAMF1, CCR2, IL33, HLA-DRB5, CCL21, LILRB1, HLA-G, CD2, ZAP70, PTPRC, IFNG, CD6, LCK, CD5, IL1B, CD27, PRKCQ, CD247, PDCD1, IL7R, HLA-DQB2, CARD11]
	innate immune response - GO:0045087	60	1.28E-31	2.00E-04	[FCN1, ITK, CIITA, NCF1, CLEC10A, SLA2, CLU, PIK3CG, JCHAIN, KYNU, LBP, ZNF683, ZBP1, GBP5, CR2, CR1, HLA-G, ZAP70, AIM2, IFNG, IFI27, LCK, XCL2, TLR8, TLR10, SMPDL3B, CLEC4E, HLA-DQB2, LTF, BLK, CCL14, GATA3, LY9, C3, CCL8, KLRK1, C7, CCL5, UBD, NLRP2, SLAMF7, SLAMF6, CCL19, CCL18, IL12RB1, HLA-DQA2, GBP1, HLA-DQA1, SLAMF1, HLA-DRB5, SIGLEC14, CCL22, CCL21, KLRC2, SH2D1A, GZMB, IFNLR1, MARCO, CD6, KLRD1]
	external side of plasma membrane - GO:0009897	40	2.07E-31	2.00E-04	[FCN1, CXCL9, TNFRSF13B, CXCR5, FASLG, IL2RG, CD3E, SPN, CD79B, CD79A, KLRK1, CD19, CXCR3, CTLA4, CCR7, CCR5, IL12RB1, ICOS, SLAMF1, LAG3, TNFRSF9, LILRB1, GP1BA, SELP, CD2, FCER2, CXCL10, PTPRC, IFNG, SELL, CD8B, CD5, CD8A, CD27, TLR8, KLRD1, PDCD1, CD69, IL7R, MS4A1]

	regulation of cell adhesion - GO:0030155	59	7.14E-29	2.00E-04	[ADAMDEC1, SIRPG, PTPN22, CD3G, CD3E, CXCL13, CD3D, PIK3CG, TNFSF13B, SPN, SIT1, TNFSF11, CTLA4, CCR7, CYTIP, HLA-DOA, CCR2, ZNF683, LAG3, ITGA4, HLA-G, LAX1, GREM1, ZAP70, IFNG, LCK, IL1B, PRKCQ, HLA-DQB2, CARD11, SKAP1, IDO1, GATA3, SPTA1, KLRK1, CCL5, ABI3BP, BTLA, CCL19, TIGIT, IL12RB1, ICOS, HLA-DQA2, GBP1, HLA-DQA1, SLAMF1, PLA2G2D, HLA-DRB5, CCL21, LILRB1, CD2, PTPRC, CD6, CD5, CAMK4, CD27, CD247, PDCD1, IL7R]
	T cell activation - GO:0042110	36	3.54E-26	2.00E-04	[ITK, RORC, PTPN22, CD3G, GATA3, ITGAL, CD1C, CD3E, SLA2, CD3D, LY9, PIK3CG, SPN, TBX21, CCR7, SLAMF6, CCL19, PLA2G2D, CCL21, BCL11B, CRTAM, THEMIS, RHOH, LILRB1, CD2, ZAP70, HSH2D, PTPRC, IFNG, CD8B, LCK, CD8A, CD7, CLEC4E, IL7R, CARD11]
**4**	chloride channel activity - GO:0005254	10	3.01E-06	0.0254	[GABRP, GLRA2, CLCN4, TTYH1, FXYD3, GABRA3, FXYD1, SLC26A4, ANO5, GABRG2]
	neurogenesis - GO:0022008	53	5.00E-06	0.0392	[ALK, ATP8A2, PPP1R9A, RND2, KIF17, HOXC10, RIMS2, GRIP1, CHL1, SOX8, PHGDH, SOX9, NEFH, TRIM67, CHRNB2, MYOC, COL25A1, OLIG1, OLIG2, GFRA3, MAG, SFRP1, OLFM3, RARB, ASPA, CRB1, NLGN1, PLPPR5, LRP4, BHLHE22, UGT8, SLITRK2, PTPRZ1, MAP2, CNR1, FLRT1, ADGRG6, SPP1, APOD, LINGO2, BCHE, KCNJ10, EYA1, CNTN6, LGI4, S100B, SORL1, MT3, LIN28A, LHX2, FABP7, FGF13, HCN1]
**5**	cell adhesion - GO:0007155	81	1.34E-28	2.00E-04	[PCDHGB7, SPON1, TENM3, TNC, HBB, ICAM5, SLC7A11, ARHGAP6, HAPLN4, HAPLN1, COMP, CDH2, ITGB8, NRCAM, EDIL3, PCDHAC2, POSTN, KIRREL2, ACTN2, APLP1, OMD, EPDR1, PCDHA13, PCDHA11, PCDHA10, CLDN11, IL1RAPL1, ADGRB1, PKP2, ITGA8, COL8A1, EPHA3, FREM2, ASTN1, PCDH10, NRXN1, NTM, NRXN3, ADAM22, NRXN2, THBS2, THBS4, COL19A1, ADD2, ACAN, NT5E, SRPX2, EFS, RELN, FLRT3, PCDHA5, PCDHA4, SPOCK1, PCDHA3, NCAM1, NCAM2, PCDHA7, PCDHA6, NLGN4Y, NLGN4X, PCDH9, ANGPT1, NEGR1, PCDH20, BMP7, PTPRD, NFASC, KRT18, PCDHB2, ITGA10, PCDHB16, PCDHB6, CNTN1, ITGBL1, PCDHB5, CNTN3, PCDHB3, CNTN4, NECTIN3, SDK2, ADGRL3]
	biological adhesion - GO:0022610	81	2.22E-28	2.00E-04	[PCDHGB7, SPON1, TENM3, TNC, HBB, ICAM5, SLC7A11, ARHGAP6, HAPLN4, HAPLN1, COMP, CDH2, ITGB8, NRCAM, EDIL3, PCDHAC2, POSTN, KIRREL2, ACTN2, APLP1, OMD, EPDR1, PCDHA13, PCDHA11, PCDHA10, CLDN11, IL1RAPL1, ADGRB1, PKP2, ITGA8, COL8A1, EPHA3, FREM2, ASTN1, PCDH10, NRXN1, NTM, NRXN3, ADAM22, NRXN2, THBS2, THBS4, COL19A1, ADD2, ACAN, NT5E, SRPX2, EFS, RELN, FLRT3, PCDHA5, PCDHA4, SPOCK1, PCDHA3, NCAM1, NCAM2, PCDHA7, PCDHA6, NLGN4Y, NLGN4X, PCDH9, ANGPT1, NEGR1, PCDH20, BMP7, PTPRD, NFASC, KRT18, PCDHB2, ITGA10, PCDHB16, PCDHB6, CNTN1, ITGBL1, PCDHB5, CNTN3, PCDHB3, CNTN4, NECTIN3, SDK2, ADGRL3]
	extracellular matrix - GO:0031012	59	8.97E-25	2.00E-04	[VIT, SPON1, CPXM2, ELN, TNC, PCSK6, HAPLN4, HAPLN1, COMP, PODNL1, FGF9, EMILIN3, COL10A1, EDIL3, TIMP4, POSTN, APLP1, OMD, P3H2, WNT16, ASPN, SFRP2, MMP13, MMP16, COL8A1, COL4A5, ANGPTL4, EMID1, FREM2, COL11A1, PTN, THBS2, THBS4, COL19A1, COCH, ACAN, RELN, FLRT3, EPYC, GPC3, SPOCK1, CILP2, NDP, GPC4, WNT2, GPC6, LRRN3, TFPI2, BMP7, MFAP5, LRFN5, CILP, SMOC1, OGN, MFAP2, COL20A1, COL9A3, LRRN1, FMOD]
	neurogenesis - GO:0022008	99	1.28E-23	2.00E-04	[STMN2, TNC, SOX2, FGF5, SALL1, CDH2, DPYSL5, KIF5C, KIF5A, NRCAM, POSTN, OMD, SOX11, ANK3, POU3F2, DKK1, ISL2, SFRP2, DOK5, ADGRB3, ADGRB1, EPHA3, NGEF, ASTN1, DLX1, NDRG4, DLX2, SHC3, DLX5, NTM, LPAR1, EFNB3, FLRT3, NPTX1, NKX2-5, NKX6-1, WNT2, PLXNA4, RAP1GAP2, NGFR, LRRN3, SYT1, BDNF, LIF, INHBA, BMP7, PTPRD, BMP2, LRFN5, GDNF, TRPV4, OGN, CNTN1, ZNF536, LRRN1, CNTN4, SDK2, SNAP25, TENM3, AREG, UCHL1, NEFL, NEFM, ZNF521, FOXD1, EDN3, GFRA1, WNT16, GFRA2, GAP43, HAND2, ALDH1A2, IL1RAPL1, DCX, SHANK1, LTK, SEMA3A, NRXN1, SEMA3B, NRXN3, ADAM22, PTN, RELN, ERBB4, EPYC, SLITRK6, SPOCK1, SLITRK5, NCAM1, CSMD3, NCAM2, WASF3, NTRK2, NLGN4X, NEGR1, VAX1, NFASC, FMOD, ADGRL3]
	anatomical structure morphogenesis - GO:0009653	115	3.80E-23	2.00E-04	[TNC, SOX2, COMP, SALL1, CDH2, BMPER, FGF9, DPYSL5, KIF5C, KIF5A, NRCAM, MYOZ2, POSTN, IGFBP5, ACTN2, OSR1, APLP1, OMD, NPY1R, SOX11, ANK3, DKK1, SFRP4, RBP4, ISL2, SFRP2, DOK5, ADGRB3, ADGRB1, PKP2, COL8A1, FREM2, DLX1, NDRG4, DLX2, SHC3, DLX5, DLX6, EFNB3, FLRT3, NDP, NPTX1, NKX2-5, NKX6-1, WNT2, PLXNA4, STRA6, NGFR, TFAP2B, LRRN3, BDNF, LIF, INHBA, BMP7, BMP2, LRFN5, GDNF, TRPV4, OGN, LRRN1, CNTN4, SDK2, THRB, CXCL8, TENM3, SYCP2, ELN, MEOX2, AREG, TMEM100, UCHL1, NEFL, COL10A1, FOXD1, GFRA1, WNT16, GFRA2, ALDH1A3, GAP43, MMP13, MMP16, HAND2, ALDH1A2, ITGA8, ANGPTL4, MDFI, SHANK1, SEMA3A, NRXN1, COL11A1, SEMA3B, KCNA2, NRXN3, PTN, HOXD11, PTGS2, SRPX2, RELN, ERBB4, EPYC, GPC3, SLITRK6, SLITRK5, NCAM1, GPC4, NTRK2, ANGPT1, EYA4, VAX1, NFASC, KRT18, WT1, MFAP2, FMOD, NECTIN3]
	axon development - GO:0061564	46	4.61E-21	2.00E-04	[SHC3, DLX5, SEMA3A, NRXN1, SEMA3B, NRXN3, TNC, UCHL1, EFNB3, RELN, FLRT3, DPYSL5, EPYC, KIF5C, KIF5A, SLITRK6, NEFL, SLITRK5, NEFM, NCAM1, NRCAM, NPTX1, NCAM2, PLXNA4, NGFR, FOXD1, LRRN3, BDNF, OMD, GFRA1, ANK3, VAX1, BMP7, GFRA2, ISL2, NFASC, LRFN5, GAP43, GDNF, DOK5, OGN, ADGRB1, LRRN1, CNTN4, FMOD, EPHA3]
	synapse organization - GO:0050808	31	5.00E-20	2.00E-04	[NRXN1, NRXN3, TNC, NRXN2, RELN, CDH2, FLRT3, LRRTM2, SLITRK6, NRCAM, NLGN4Y, NTRK2, NLGN4X, BDNF, ANK3, DKK1, PTPRD, NFASC, GDNF, GLRB, PCDHB2, ADGRB3, IL1RAPL1, PCDHB16, PCDHB6, COL4A5, PCDHB5, PCDHB3, SDK2, ADGRL3, SHANK1]
	regulation of nervous system development - GO:0051960	60	2.03E-17	2.00E-04	[SNAP25, TENM3, STMN2, SOX2, CDH2, LRRTM2, NEFL, NRCAM, SOX11, DKK1, POU3F2, ISL2, SFRP2, ADGRB3, IL1RAPL1, ADGRB1, EPHA3, NGEF, SHANK1, DLX1, LTK, NDRG4, DLX2, SEMA3A, NRXN1, LPAR1, NRXN3, PTN, THBS2, SRPX2, RELN, FLRT3, ERBB4, SLITRK6, SPOCK1, SLITRK5, CSMD3, NKX2-5, NKX6-1, WNT2, WASF3, PLXNA4, RAP1GAP2, NGFR, NTRK2, LRRN3, NEGR1, SYT1, BDNF, LIF, VAX1, BMP7, PTPRD, BMP2, TRPV4, CNTN1, ZNF536, LRRN1, CNTN4, ADGRL3]
	cell morphogenesis involved in neuron differentiation - GO:0048667	42	3.78E-17	2.00E-04	[SHC3, DLX5, SEMA3A, NRXN1, SEMA3B, NRXN3, UCHL1, EFNB3, RELN, FLRT3, DPYSL5, EPYC, KIF5C, KIF5A, SLITRK6, SLITRK5, NCAM1, NRCAM, NPTX1, PLXNA4, NGFR, FOXD1, LRRN3, BDNF, OMD, GFRA1, ANK3, VAX1, BMP7, GFRA2, ISL2, NFASC, LRFN5, GAP43, GDNF, DOK5, OGN, ADGRB1, LRRN1, CNTN4, FMOD, SHANK1]
	neuron projection morphogenesis - GO:0048812	44	4.84E-17	2.00E-04	[SHC3, DLX5, SEMA3A, NRXN1, SEMA3B, NRXN3, UCHL1, EFNB3, RELN, FLRT3, DPYSL5, EPYC, KIF5C, KIF5A, SLITRK6, NEFL, SLITRK5, NCAM1, NRCAM, NPTX1, PLXNA4, NGFR, POSTN, FOXD1, LRRN3, BDNF, OMD, GFRA1, ANK3, VAX1, BMP7, GFRA2, ISL2, NFASC, LRFN5, GAP43, GDNF, DOK5, OGN, ADGRB1, LRRN1, CNTN4, FMOD, SHANK1]

Table S2: Gene ontology enrichments for the five gene clusters. Top 10 significant GO terms are mentioned for each cluster. Enrichments were calculated using TANGO[4].

Gene Cluster	KEGG Pathway	#genes	Raw p-value	Corrected p-value	Enrichment factor	Gene list
1	Melanogenesis	9	7.25E-05	0.00414	5.07	[GNAO1, DCT, KIT, TYRP1, FZD9, ADCY2, ADCY1, TYR, WNT4]
	Calcium signaling pathway	10	0.00119	0.0442	3.22	[RYR1, CHRM1, GNAL, CACNA1B, ATP2B3, CACNA1D, ADCY2, ADCY1, MYLK3, CACNA1H]
	Maturity onset diabetes of the young	4	8.83E-04	0.0357	9.11	[PKLR, ONECUT1, MNX1, NKX2-2]
3	Natural killer cell mediated cytotoxicity	17	3.07E-11	3.52E-09	8.19	[KLRC2, PRKCB, SH2D1A, PRF1, GZMB, FASLG, ITGAL, HLA-G, KIR2DL4, PIK3CG, ZAP70, KLRK1, IFNG, LCK, KLRD1, CD48, CD247]
	Graft-versus-host disease	12	6.87E-13	9.53E-11	19.2	[HLA-DRB5, IFNG, IL1B, PRF1, GZMB, FASLG, KLRD1, HLA-DOA, HLA-DQA2, HLA-G, HLA-DOB, HLA-DQA1]
	B cell receptor signaling pathway	8	1.87E-05	0.00129	6.99	[CD79B, CD79A, CR2, PRKCB, CD19, CARD11, CD22, PIK3CG]
	Allograft rejection	10	1.44E-10	1.39E-08	17.7	[HLA-DRB5, IFNG, PRF1, GZMB, FASLG, HLA-DOA, HLA-DQA2, HLA-G, HLA-DOB, HLA-DQA1]
	Primary immunodeficiency	14	4.83E-17	1.56E-14	26.2	[CIITA, TNFRSF13B, IL2RG, CD3E, CD3D, CD79A, ZAP70, PTPRC, CD8B, LCK, CD8A, CD19, IL7R, ICOS]
	Leukocyte transendothelial migration	8	3.87E-04	0.0171	4.56	[ITK, NCF1, ITGA4, PRKCB, RHOH, ITGAL, MMP9, PIK3CG]
	Hematopoietic cell lineage	21	1.12E-19	5.41E-17	15.8	[CR2, HLA-DRB5, CR1, ITGA4, CD3G, GP1BA, CD1C, CD3E, CD3D, CD2, FCER2, CD8B, CD5, CD8A, IL1B, CD19, CD7, CD38, IL7R, MS4A1, CD22]
	Autoimmune thyroid disease	10	5.32E-09	4.70E-07	12.6	[HLA-DRB5, PRF1, GZMB, CTLA4, FASLG, HLA-DOA, HLA-DQA2, HLA-G, HLA-DOB, HLA-DQA1]
	Type I diabetes mellitus	11	3.27E-11	3.52E-09	16.8	[HLA-DRB5, IFNG, IL1B, PRF1, GZMB, FASLG, HLA-DOA, HLA-DQA2, HLA-G, HLA-DOB, HLA-DQA1]
	Chemokine signaling pathway	22	1.59E-13	3.08E-11	7.63	[CCL14, ITK, CXCL9, CCL22, CCL21, NCF1, PRKCB, CXCR5, CXCR6, CXCL13, PIK3CG, CXCL10, CXCL11, CCL8, CCL5, CXCR3, XCL2, CCR7, CCL19, CCL18, CCR5, CCR2]
	Cytokine-cytokine receptor interaction	35	6.30E-23	6.11E-20	8.79	[CCL14, CXCL9, TNFRSF13B, CXCR5, FASLG, TNFRSF11B, CXCR6, IL2RG, CXCL13, TNFSF13B, CCL8, CCL5, CXCR3, IL21R, TNFRSF17, TNFSF11, CCR7, CCL19, CCL18, CCR5, IL12RB1, CCR2, CCL22, CCL21, CD70, TNFRSF9, IFNLR1, CXCL10, CXCL11, IFNG, IL1B, XCL2, CD27, LTB, IL7R]
	Asthma	5	8.35E-05	0.0045	10.9	[HLA-DRB5, HLA-DOA, HLA-DQA2, HLA-DOB, HLA-DQA1]
	Toll-like receptor signaling pathway	8	1.59E-04	0.00771	5.19	[CXCL10, CXCL11, CXCL9, CCL5, IL1B, TLR8, LBP, PIK3CG]
	Systemic lupus erythematosus	8	0.00117	0.0442	3.85	[C3, HLA-DRB5, IFNG, C7, HLA-DOA, HLA-DQA2, HLA-DOB, HLA-DQA1]
	Cell adhesion molecules (CAMs)	22	7.20E-17	1.74E-14	10.9	[CADM3, HLA-DRB5, ITGA4, ITGAL, SELE, HLA-G, CD2, SELP, SPN, PTPRC, CD6, CD8B, SELL, CD8A, CTLA4, PDCD1, HLA-DOA, ICOS, HLA-DQA2, HLA-DOB, HLA-DQA1, CD22]
	Antigen processing and presentation	12	9.42E-09	7.62E-07	8.94	[CIITA, HLA-DRB5, CD8B, KLRC2, CD8A, KLRD1, HLA-DOA, HLA-DQA2, HLA-G, HLA-DOB, HLA-DQA1, KIR2DL4]
	T cell receptor signaling pathway	17	6.73E-13	9.53E-11	10.3	[ITK, CD3G, CD3E, CD3D, PIK3CG, ZAP70, PTPRC, CD8B, IFNG, CD8A, LCK, CTLA4, PRKCQ, CD247, PDCD1, ICOS, CARD11]
5	Arrhythmogenic right ventricular cardiomyopathy (ARVC)	9	4.28E-05	0.00259	5.38	[DES, CDH2, ACTN2, CACNA2D1, ITGA10, PKP2, ITGA8, ITGB8, CACNG4]
	Neuroactive ligand-receptor interaction	16	2.54E-04	0.0117	2.77	[GABRA2, GRIA1, CHRM3, GRIA2, THRB, LPAR1, NPY1R, ADRB1, GRIK2, PRLR, MCHR1, GHR, GABRR1, GLRB, F2RL2, NTSR1]
	ECM-receptor interaction	9	1.17E-04	0.00596	4.74	[COMP, RELN, COL11A1, ITGA10, TNC, ITGA8, ITGB8, THBS2, THBS4]
	Dilated cardiomyopathy	10	3.59E-05	0.00232	4.92	[PLN, DES, CACNA2D1, ITGA10, ITGA8, ITGB8, ADRB1, ADCY8, CACNG4, ADCY5]
	Cell adhesion molecules (CAMs)	15	3.14E-07	2.34E-05	5.03	[NLGN4X, NEGR1, NRXN1, NRXN3, NRXN2, CLDN11, NFASC, CDH2, CNTN1, ITGA8, ITGB8, NRCAM, NCAM1, NCAM2, NECTIN3]
	TGF-beta signaling pathway	8	7.28E-04	0.0307	4.12	[COMP, BMP2, FST, BMP8B, INHBA, THBS2, BMP7, THBS4]

Table S3: Enrichment analysis for KEGG pathways performed using PROMO on the five gene clusters. Top significant KEGG pathways are displayed for each gene cluster. Enrichments were calculated using TANGO[4].

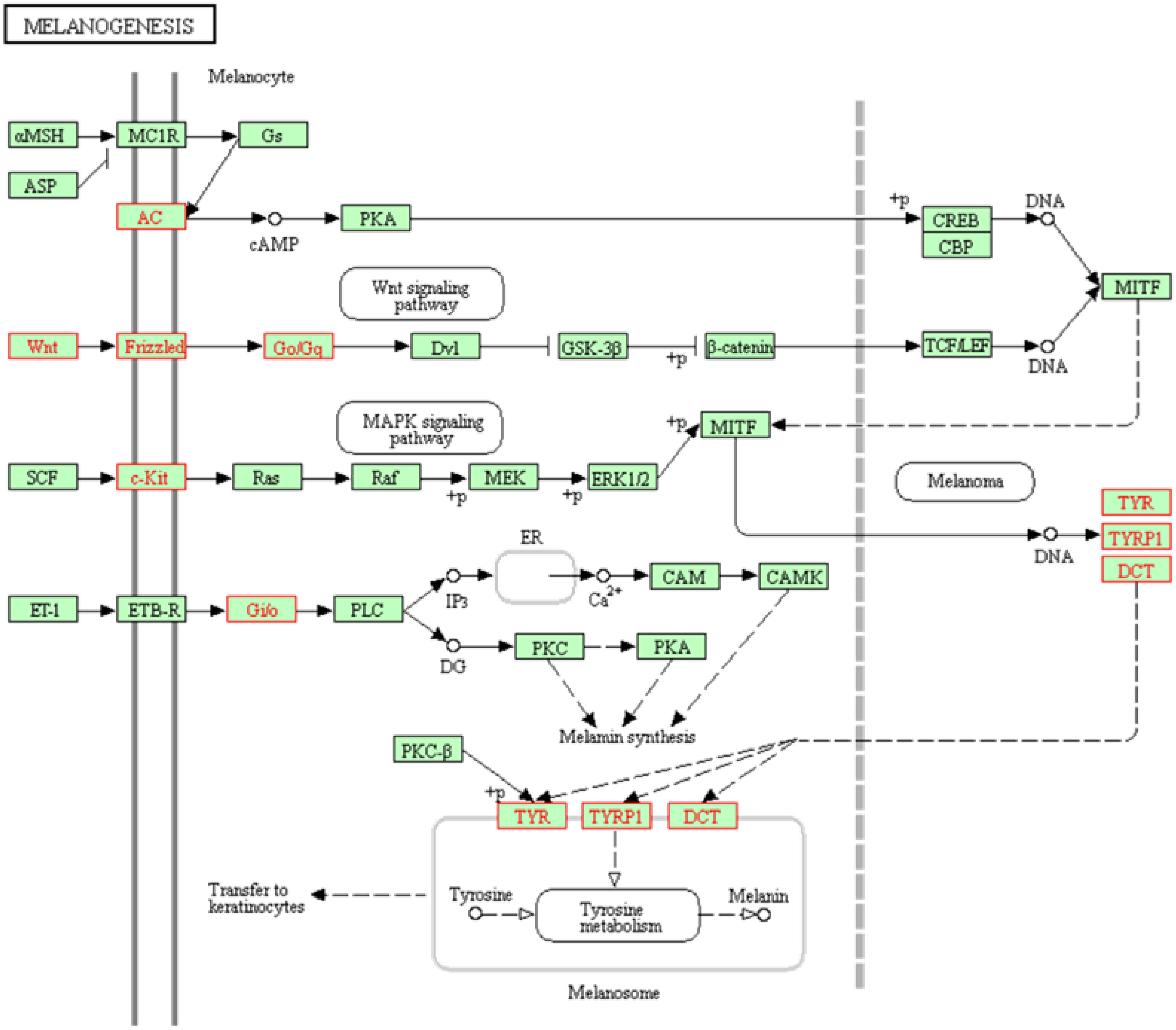

Figure S4: Sample-cluster 4 (the 'Melanogenesis-high' cluster) overexpressed genes in the KEGG "Melanogenesis" pathway. The 384 genes composing cluster 1 (Fig. 1a) are overexpressed in the 'Melanogenesis-high' sample cluster and were found to be enriched (p<0.005) for the Melanogenesis KEGG pathway. The nine Melanogenesis pathway genes (TYR, WNT4, KIT, DCT, FZD9, GNAO1, ADCY1, ADCY2, TYRP1) are marked in in red. The analysis was performed using the Expander[3,4] tool. Pathway and graphics were taken from the Kyoto Encyclopedia of Genes and Genomes (KEGG[5]) database.

Id	Gene Symbol	p-value	FC	Id	Gene Symbol	p-value	FC
**1**	**OCA2**	7.79E-11	5.72	**51**	DUSP9	1.31E-09	1.70
**2**	**TYRP1**	7.12E-08	5.62	**52**	WNK2	5.49E-06	1.67
**3**	ITGB1BP3	9.04E-13	3.29	**53**	CDK2	1.56E-14	1.66
**4**	SLC7A4	1.07E-06	3.03	**54**	SEPT3	5.12E-11	1.66
**5**	IP6K3	9.92E-06	2.94	**55**	LAMA1	6.79E-06	1.66
**6**	C14orf34	4.50E-08	2.78	**56**	KCNAB2	3.08E-13	1.62
**7**	GABRA5	5.46E-10	2.67	**57**	MGC16025	6.77E-13	1.62
**8**	KRTAP19-1	8.80E-12	2.66	**58**	PRR5-ARHGAP8	5.81E-05	1.61
**9**	FAM69C	4.69E-16	2.64	**59**	SNCB	6.44E-06	1.60
**10**	ABCB5	7.16E-12	2.64	**60**	GNAO1	1.08E-07	1.58
**11**	**KIT**	3.97E-07	2.43	**61**	LOC148145	1.92E-05	1.57
**12**	VGF	2.12E-05	2.41	**62**	MAST1	4.67E-07	1.56
**13**	SLC6A17	4.06E-12	2.29	**63**	HRK	2.00E-07	1.56
**14**	MGAT5B	5.10E-14	2.29	**64**	PLAC2	3.70E-05	1.54
**15**	GNAL	2.21E-09	2.22	**65**	C6orf176	6.07E-09	1.52
**16**	ACCSL	7.46E-11	2.22	**66**	SFTPC	1.36E-07	1.50
**17**	ABCC2	5.53E-09	2.18	**67**	RIMS4	3.04E-06	1.49
**18**	ONECUT1	2.98E-09	2.16	**68**	ONECUT2	2.86E-05	1.48
**19**	NECAB2	7.78E-09	2.16	**69**	FZD9	5.88E-09	1.48
**20**	CNTFR	2.62E-05	2.13	**70**	ARHGAP8	3.11E-05	1.47
**21**	PRODH	1.47E-07	2.09	**71**	LOC100127888	2.29E-09	1.47
**22**	TRPM1	5.16E-12	2.07	**72**	**TRIM63**	4.41E-16	1.46
**23**	PNMA6A	1.03E-16	2.06	**73**	DGCR5	7.95E-06	1.45
**24**	POU4F1	2.54E-07	2.02	**74**	EPHA5	1.94E-07	1.45
**25**	SLC5A10	9.72E-07	1.97	**75**	TMEM151A	1.11E-05	1.42
**26**	**SILV**	1.57E-15	1.97	**76**	NRTN	1.72E-05	1.40
**27**	FOXF2	2.70E-09	1.95	**77**	GBX2	7.23E-05	1.40
**28**	SLC16A6	4.45E-05	1.91	**78**	C1QL4	2.60E-08	1.40
**29**	CDK15	2.27E-07	1.91	**79**	FSTL4	1.70E-06	1.40
**30**	L1CAM	1.84E-06	1.89	**80**	CPNE7	2.24E-06	1.39
**31**	CDH3	1.03E-10	1.88	**81**	DUSP8	1.86E-08	1.39
**32**	DPYSL4	1.42E-10	1.87	**82**	TFAP2A	8.93E-22	1.38
**33**	KIF1A	5.17E-06	1.87	**83**	C6orf218	4.23E-12	1.35
**34**	NKX2-8	6.01E-05	1.87	**84**	ZNF703	4.07E-14	1.34
**35**	BRSK2	2.79E-09	1.86	**85**	HES6	5.65E-08	1.33
**36**	PITX2	6.27E-05	1.85	**86**	C15orf59	7.46E-05	1.31
**37**	PRRT4	7.56E-07	1.85	**87**	LGI3	1.28E-05	1.30
**38**	ADAM11	2.01E-11	1.82	**88**	NCRNA00052	2.77E-07	1.30
**39**	MCF2L	5.82E-09	1.82	**89**	TPCN2	1.73E-10	1.28
**40**	RTN4R	1.03E-14	1.81	**90**	LOC390595	9.71E-06	1.27
**41**	CA14	3.96E-14	1.80	**91**	ADAMTSL5	1.22E-07	1.27
**42**	NR4A3	8.93E-10	1.80	**92**	**DCT**	6.46E-05	1.27
**43**	TSPAN10	1.03E-11	1.78	**93**	GPRC5A	6.85E-05	1.26
**44**	TPPP	1.09E-11	1.77	**94**	BAIAP2L1	1.57E-09	1.26
**45**	GMPR	1.43E-12	1.77	**95**	ANKRD9	7.20E-13	1.25
**46**	KCNH1	1.07E-06	1.76	**96**	ITPKB	6.43E-11	1.25
**47**	DLL3	1.41E-09	1.74	**97**	TTYH2	1.51E-13	1.25
**48**	KREMEN2	2.50E-08	1.74	**98**	CELF5	2.06E-05	1.25
**49**	SEMA6A	5.63E-20	1.72	**99**	MANEAL	4.53E-07	1.25
**50**	SULT4A1	5.47E-05	1.72	**100**	LRRC39	7.89E-07	1.24

Table S4: List of the 100 most over-expressed genes distinguishing cluster 4 samples from all other clusters. Genes are sorted by descending mean fold-change(FC). P-value was calculated using the rank-sum test

applied on [Melanogenesis-high] samples (n=118) vs. [Immune,Keratin,Melanogenesis-low] samples (n=351). p-value cutoff: p<0.0001. Genes belonging to the 'Melanin biosynthesis' GO term were identified using the GORILLA[12] tool and are marked in bold. Complete lists of overexpressed genes, in addition to overexpressed genes in the other sample clusters, appear on Additional File 2.

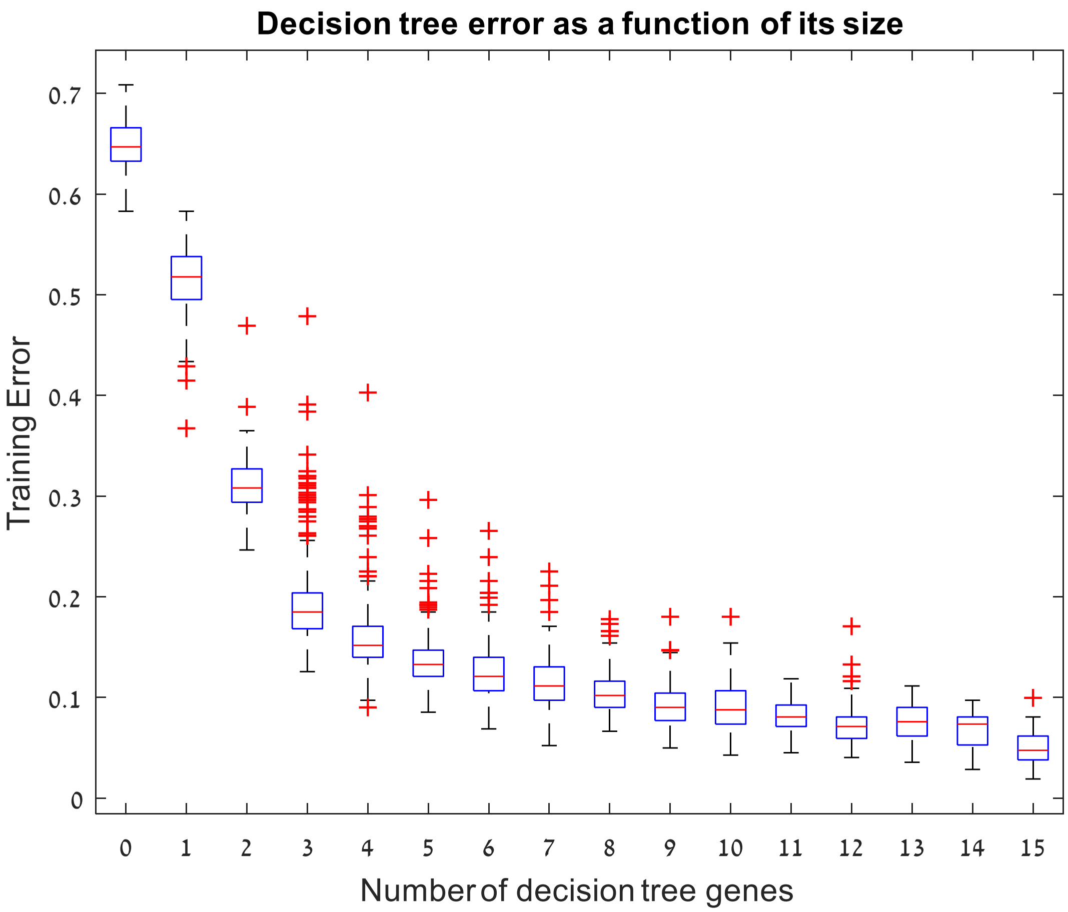

Figure S5: Error of decision tree classifiers as a function of the number of predictor genes. For a varying number of genes (1-15) and for varying pruning levels (0-10), 30 decision trees were trained on resampled subsets of the dataset samples (resampling ratio of 0.9). The graph shows the average training error for each decision tree size. A three-gene classifier for predicting melanoma's molecular subtype gives a good balance between simplicity (avoiding over-fitting) and performance and reaches a training error that is reasonably close to that obtained by a larger number of genes.

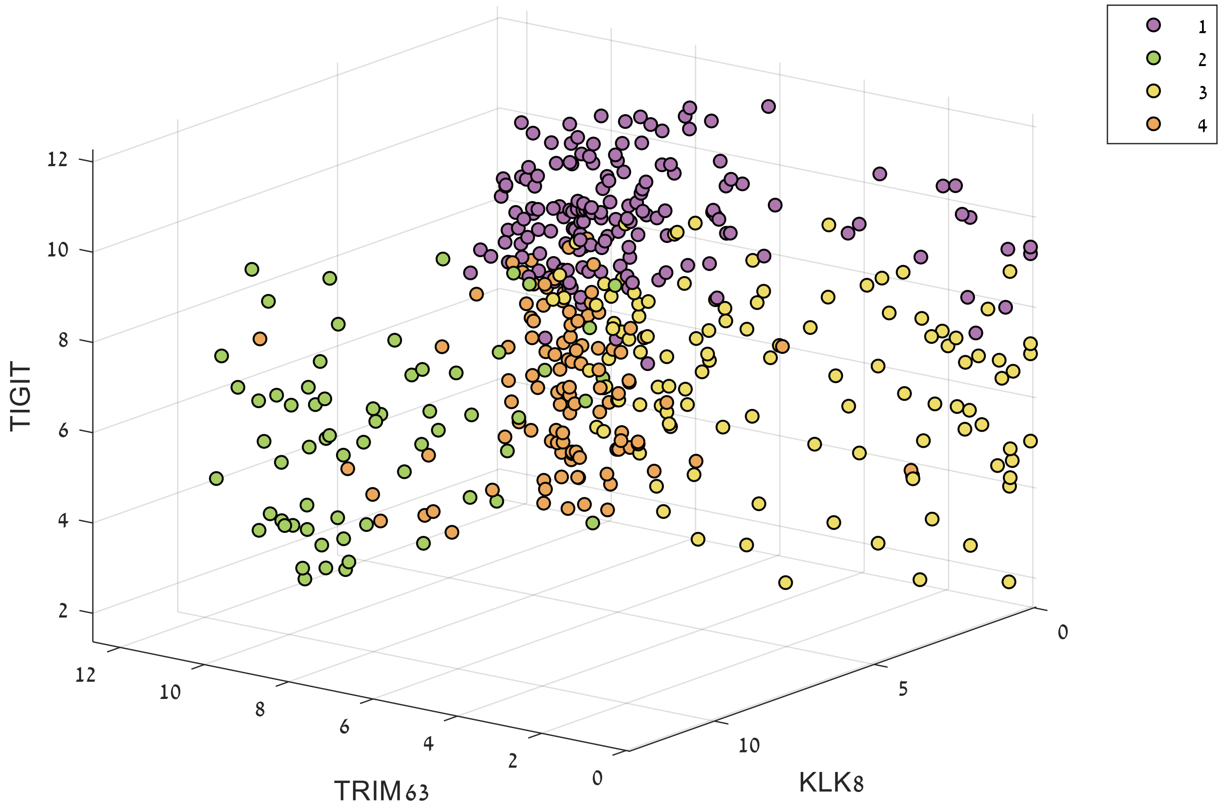

Figure S6: Dispersion of the 469 melanoma samples projected to the 3-dimensional space of the three selected classifier predictors: KLK8, TIGIT, and TRIM63. Samples are colored by the melanoma subgroups. The axis representing the expression level of the KLK gene distinguishes cluster 2 samples (green circles, “Keratin” subgroup) showing high levels of KLK8 expression, from all other clusters. The axis representing the expression of the TIGIT gene distinguishes cluster 1 samples (purple circles, “Immune” subgroup) showing high levels of TIGIT expression, from the other subgroups. Lastly, the axis representing the expression of the TRIM63 gene distinguished cluster 3 samples (yellow circles, “Melanogenesis-low” subtype) from cluster 4 samples (orange circles, “Melanogenesis-high” subtype).

KLK8 (https://www.proteinatlas.org/ENSG00000129455-KLK8/tissue)

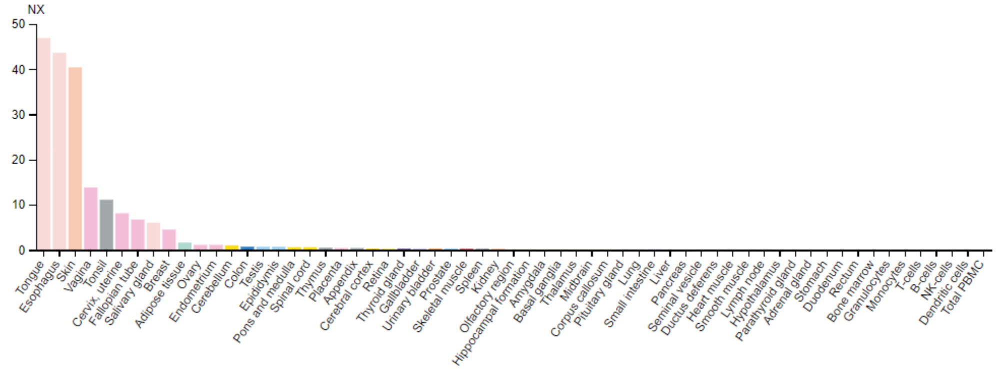

TIGIT (https://www.proteinatlas.org/ENSG00000181847-TIGIT/tissue)

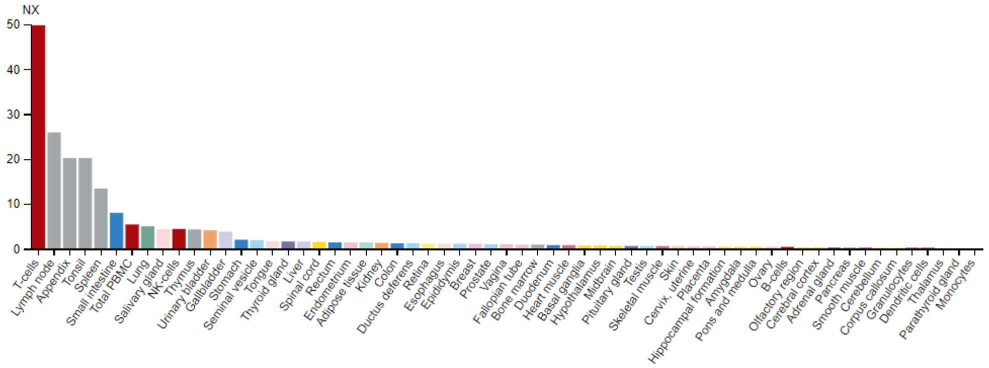

TRIM63 (https://www.proteinatlas.org/ENSG00000158022-TRIM63/tissue)

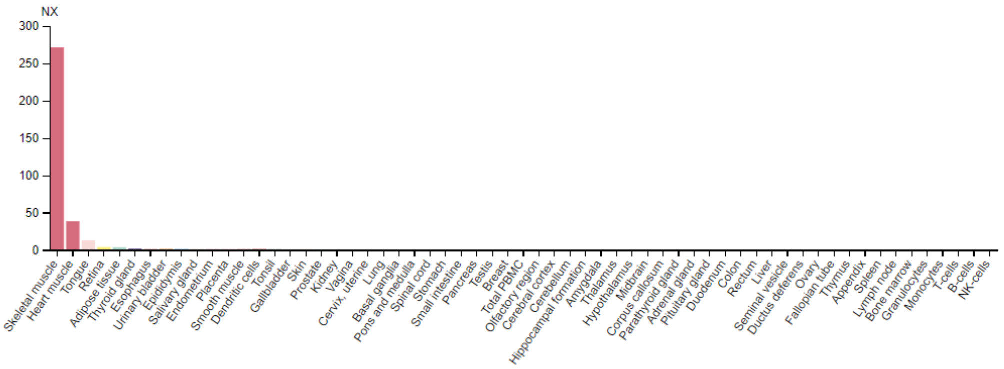

Figure S7: The RNA expression levels on normal tissues of the three classifier predictors: KLK8, TIGIT, and TRIM63. Consensus Normalized eXpression (NX) levels for 55 tissue types and six blood cell types, created by The Human Protein Atlas[6,7] by combining the data from three transcriptomics datasets (HPA[6], GTEx[8] and FANTOM5[9,10,11]). KLK8 is primarily expressed in tongue, esophagus and skin tissues. TIGIT is primarily expressed in blood (T-cells) and lymphoid tissues. TRIM63 is primarily expressed in skeletal muscle tissue. Source: Human Protein Atlas available from http://www.proteinatlas.org.

Supplementary Information - Section 2

Analysis of the topology and predictor biological function of decision trees for subsampled datasets

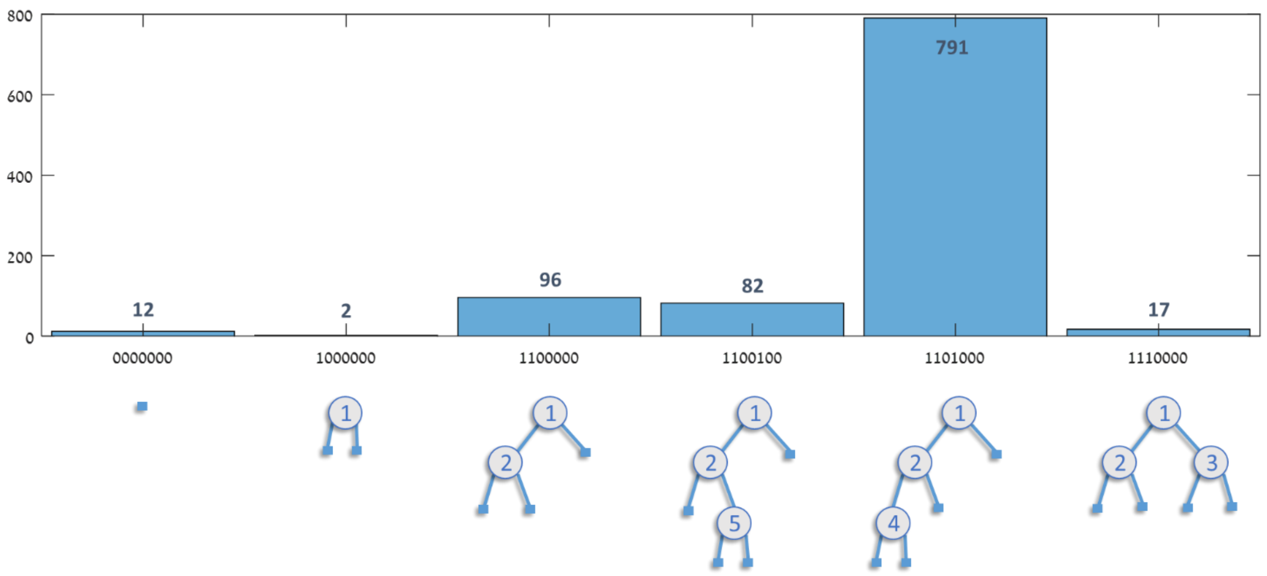

Figure S8: Distribution of the topology of 1000 decision trees. For analyzing the topology and biological function of the tree predictors, we trained 1000 3-gene decision trees by resampling the dataset samples (resample factor = 0.8). The most frequent topology was '1101000', identical to the topology of the final decistion tree presented in Fig. 4, which was trained on the entire dataset (Note that 1101000 and 1100100 are considered different since the left child of every node always corresponds to the "less than" subgroup.)

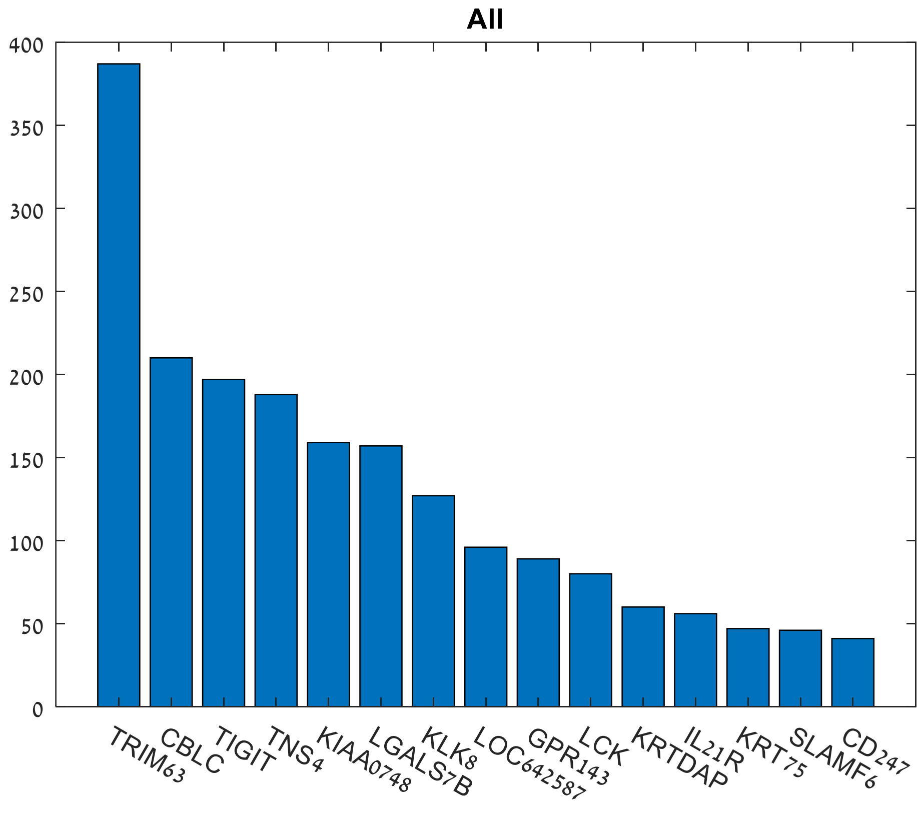

Figure S9: Most frequent genes in the 1000 decision trees (in all tree positions).

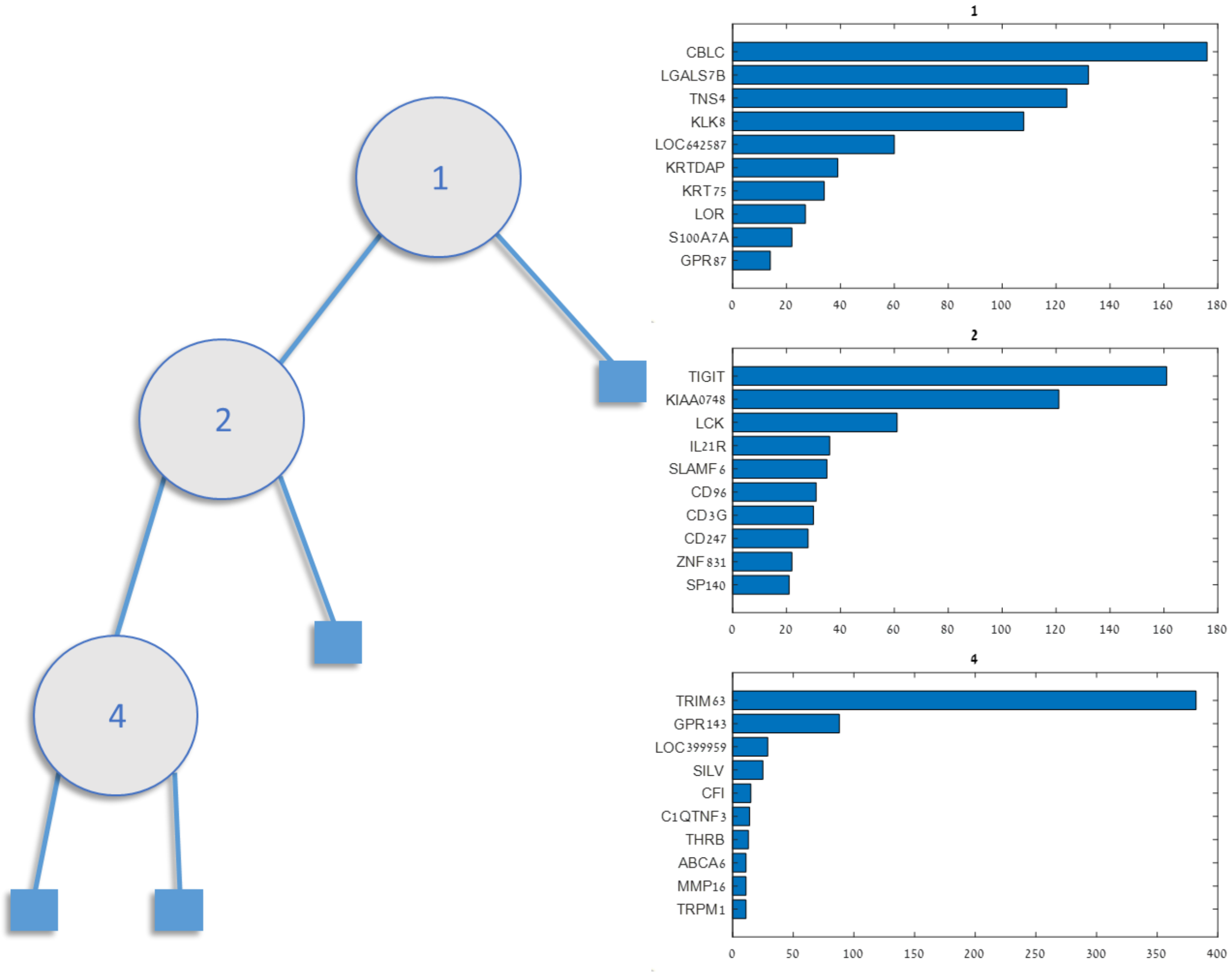

Figure S10: Most frequent predictor genes for each position in the tree and their biological function. For each position in the 1101000 topology, we generated a list of the 10 most frequent predictor genes as appearing on the 791 random tree variants. The analysis showed that the most frequent genes in each position are characterized by a specific biological function. Position 1, which forms the tree's root, was typically assigned with keratin and other skin related biomarkers, such as LGALS7B, TNS4 and KLK8. Position 2 was typically assigned with well known immune markers such as TIGIT, KIAA0748, LCK, and IL21R. Position 4 was preferentially assigned with TRIM63, but also with typical melanogenesis genes such as GPR143, SILV, and TRPM1. Interestingly, the lists also included genes that are less familiar in their context here, such as LOC399959.

The results demonstrate the hierarchy of the biological functions by which melanoma samples can be partitioned into distinct subgroups, and also show that the final tree presentd in Fig. 4 is a representative of a stable tree topology and is using predictor genes that are biomarkers of the above three biological functions.

Supplementary Information - Section 3

Experimental validation of the predictor genes

<table>
<tr><th>Patient number in Figure</th><th>Age at time of primary tumor diagnosis</th><th>Survival from T diagnosis (months)</th><th>Survival from regional lymph node (N) diagnosis (months)</th><th>Survival from distant metastasis (M) diagnosis (months)</th><th>Current status</th></tr>
<tr><td>1</td><td>81</td><td colspan="3">over 60 months</td><td>Alive</td></tr>
<tr><td>2</td><td>67</td><td colspan="3">over 60 months</td><td>Alive</td></tr>
<tr><td>3</td><td>66</td><td colspan="3">over 60 months</td><td>Alive</td></tr>
<tr><td>4</td><td>88</td><td>24</td><td>4.15</td><td></td><td>Deceased</td></tr>
<tr><td>5</td><td>74</td><td>19.75</td><td>6.77</td><td></td><td>Deceased</td></tr>
<tr><td>6</td><td>67</td><td>19.48</td><td>17.38</td><td>0.85</td><td>Deceased</td></tr>
</table>

Table S5: Clinical details for the six patients selected for Immunohistochemical staining. Patients 1-3 survived for more than 60 months after diagnosis and were therefore labeled as "Good Survival", whereas patients 4-6 survived for less than 24 months and were therefore labeled as "Poor Survival".

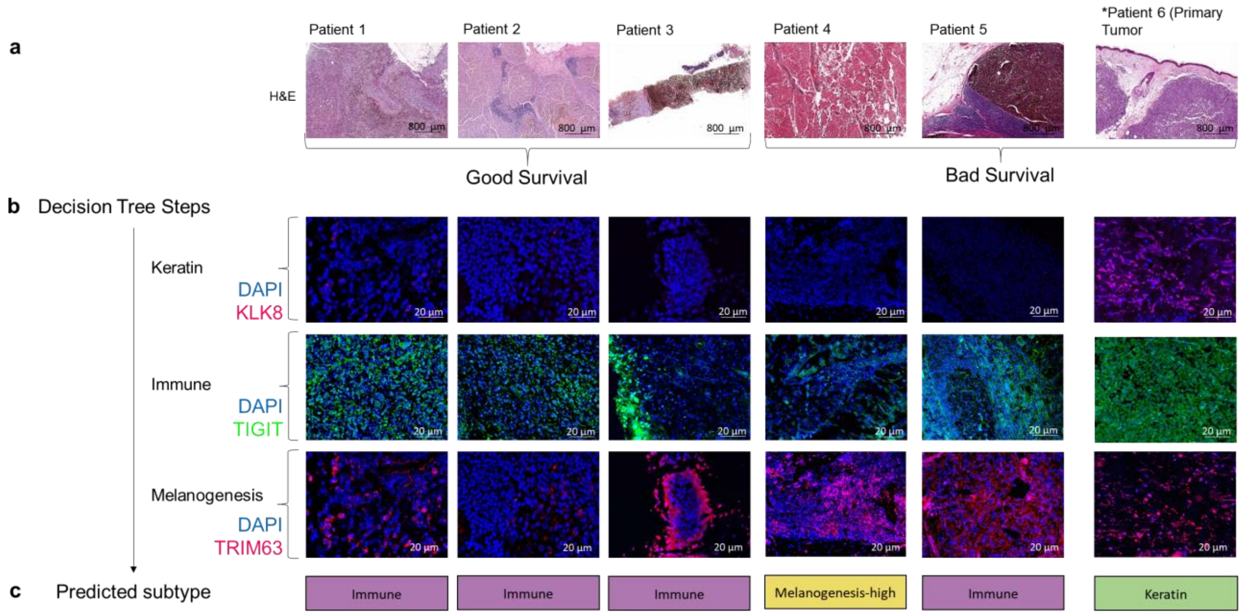

d

	Good Survival			Bad Survival			Threshold
	**1**	**2**	**3**	**4**	**5**	**6**	
**KLK8**	0.74	1.64	1.19	1.02	0.66	**5.28**	3.00
**TIGIT**	**2.59**	**1.85**	**1.62**	1.07	**2.57**	**3.13**	1.20
**TRIM63**	3.50	1.87	5.02	**9.18**	**11.18**	4.30	6.00

Figure S11: Keratin, Melanogenesis and immune characteristics of six additional samples. (a) Hematoxylin and Eosin (H&E) staining of five lymph node samples containing melanoma metastases (patients 1-5) and one primary melanoma (patient 6) from six different patients imaged at 20x magnification. Patients clinical details appear on Table S1.6. **(b)** Immunohistochemical staining of the three proteins of the decision tree on the samples of the six patients. Nuclei were stained blue using DAPI. Row 1: Using KLK8 (pink) as a predictor for the Keratin subgroup. Row 2: Using TIGIT (green) as a predictor of the Immune subgroup. Row 3: Using TRIM63 (red) as a predictor of the Melanogenesis-high subgroup. **(c)** The assignment of the specimens from the six patients to subtypes based on the expression levels of the three predictor genes are shown at the bottom bar (see more details on the next paragraph). **(d)** A matrix quantifying the fluorescence intensity of immunohistochemistry across biomarkers and patients. For each protein, an expression threshold was manually defined and over expressed values were marked in bold.

Each sample was assigned to a melanoma subgroup based on the decision tree's logic: The KLK8 expression levels distinguished sample 6, the only primary sample, from all other samples, and assigned it to the Keratin subgroup. The high expression levels of the immune marker TIGIT on samples 1-3 and 5 assigned them to the Immune subgroup. Finally, the high levels of the Melanogenesis marker TRIM63 assigned sample 4 to the Melanogenesis-high subgroup. The prediction procedure correctly identified sample 6 to keratin, and samples 1-4 to melanoma subgroups that are in agreement with their evaulated survival category . However, sample 5 was assigned to the Immune subgroup, which does not match its evaluated survival category.

Patient number on Figure	Age at time of primary tumor diagnosis	Survival from T diagnosis (months)	Survival from regional lymph node (N) diagnosis (months)	Survival from distant metastais (M) diagnosis (months)	Current status	Category determined by expert evaluation of the patient
**1**	78	45	24	6	Deceased	Good
**2**	43	47	49		Alive	Good
**3**	68	26	26		Alive	Good
**4**	90	16.4	3.85		Deceased	Bad
**5**	66	34	21		Deceased	Bad
**6**	90	27.7	22.44	24.51	Deceased	Bad

Table S6: Clinical details for the second batch of patients selected for Immunohistochemical staining. Unlike the first batch, the survival times were not sufficiently distinctive and therefore the categorization of the patients into good and bad survival was done by the expert physician.

Supplementary Information - Section 4

Comparison of the identified melanoma subgroups to the Lund subtypes

In 2010, Jönsson et al. identified four expression-based subgroups (a.k.a the Lund subgroups) by analyzing 57 stage IV melanomas taken from patients[13]. The subtypes were later confirmed on additional patient cohorts[14,15,16]. Here, we compare our melanoma subgroups to the Lund subtypes on the TCGA dataset, using labels obtained from the authors of [16]. The comparison showed that the two classifications are overall similar (Chi-square $p<3.0e-67$, see Fig. S12). Two of our subgroups, Keratin and Melanogenesis-low, showed higher similarity to the Lund subtypes Normal and Proliferative, respectively. However, the Immune and Melanogenesis-high subgroups showed lower similarity to the Lund corresponding subgroups (High immune and Pigmentation, respectively). Furthermore, our classification stratified the metastatic patients better into prognostic subgroups in terms of five-year survival (Fig. S15).

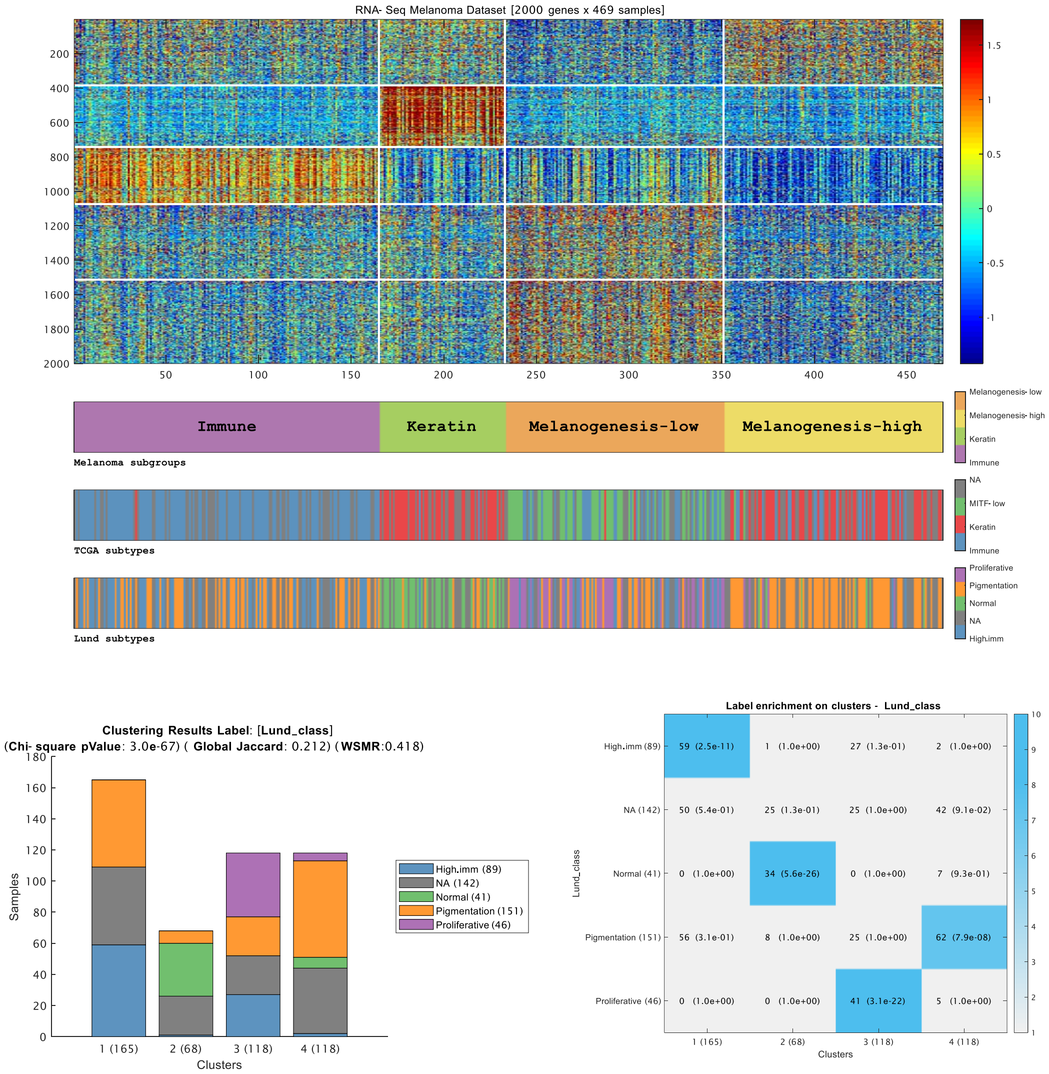

Figure S12: Comparison of the melanoma subgroups with the Lund subtypes using all dataset samples (n=469).

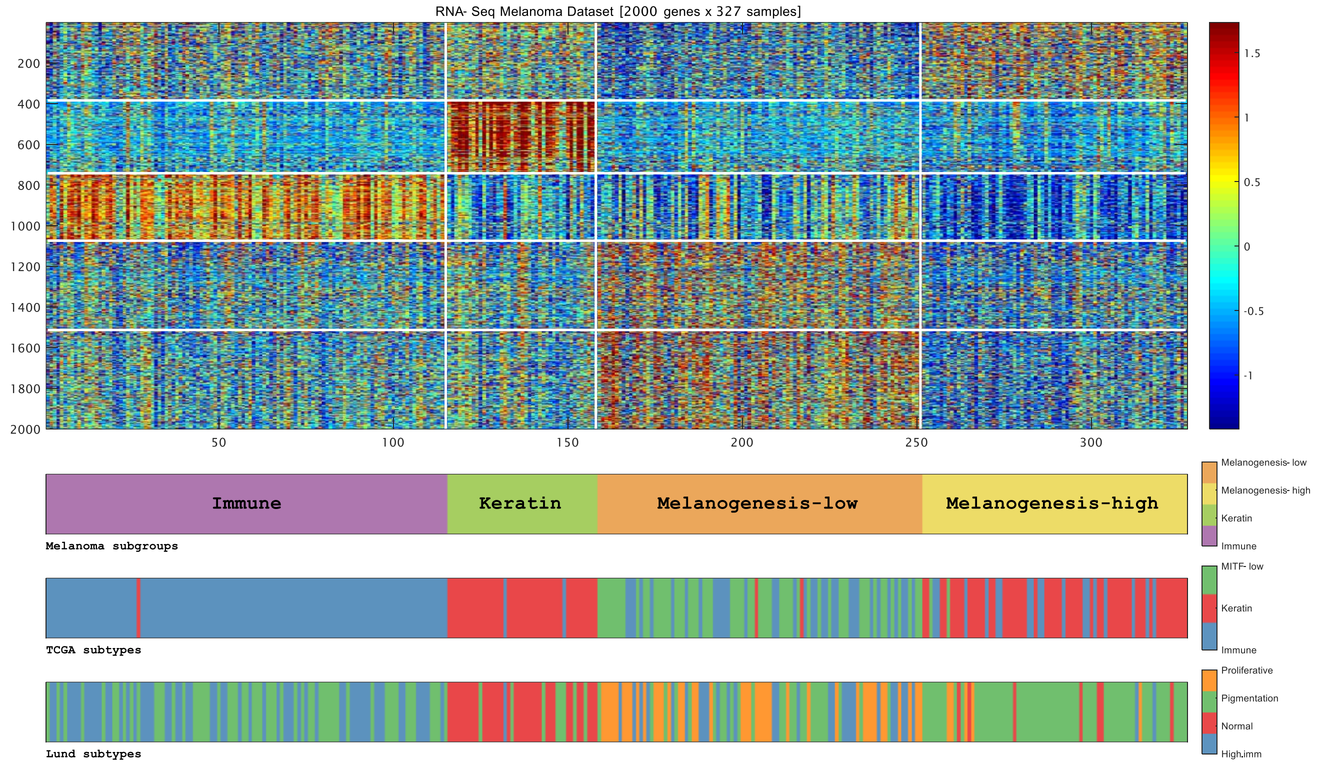

Figure S13: Comparison of the melanoma subgroups with the Lund subtypes, omitting samples for which Lund subtype is not available (n=327).

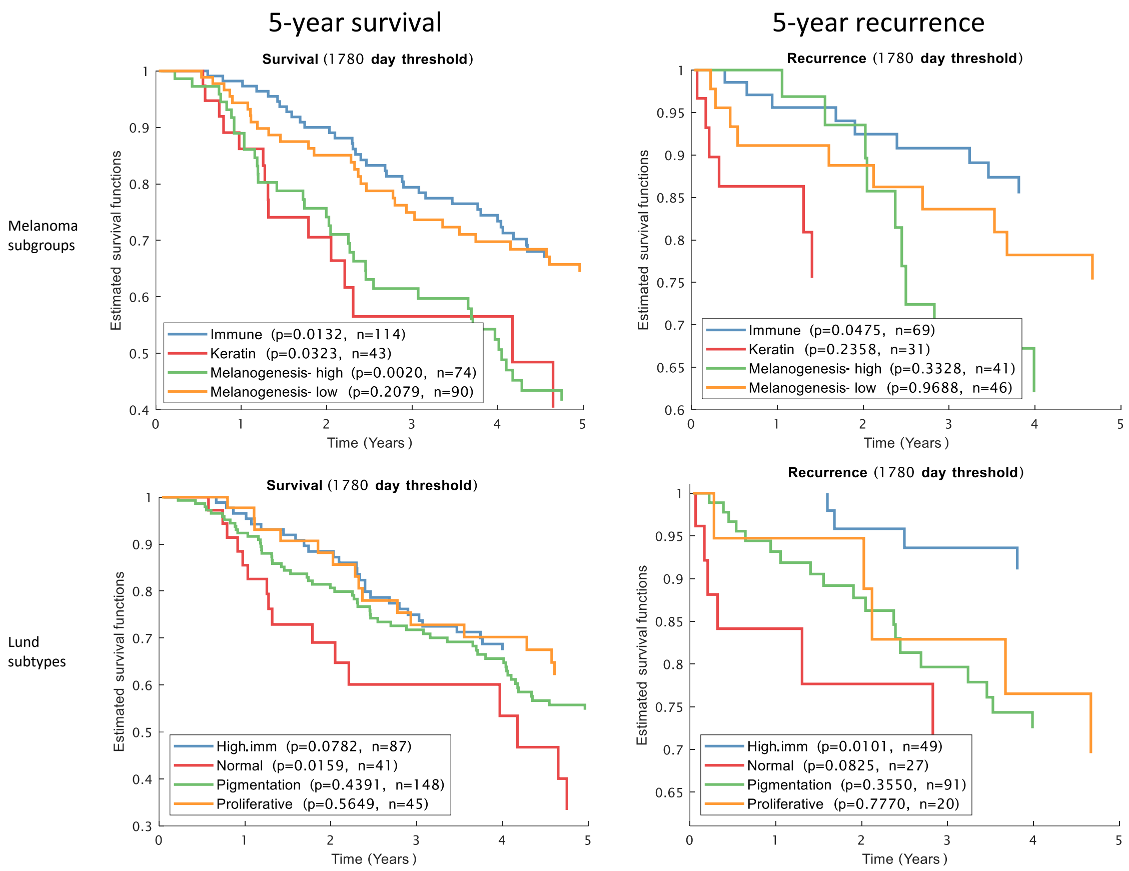

Figure S14: Comparison of five-year survival and recurrence between the melanoma subgroups identified in this study and the Lund subgroups. Only samples for which Lund subtype is available were included in the analysis (n=327).

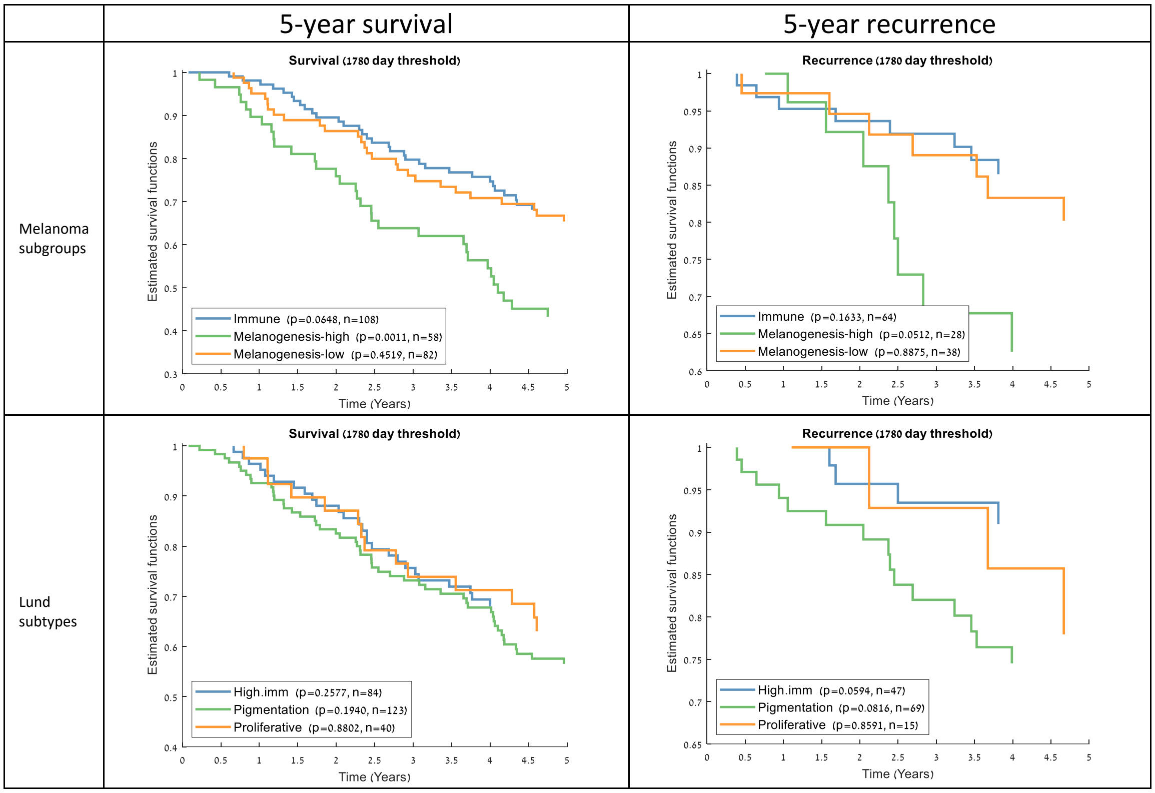

Figure S15: Five-year survival and recurrence plots for metastasis samples using the melanoma subgroups identified in this study and the Lund subgroups. Only metastasis samples for which Lund subtype and followup information are available were included in the analysis. Analysis was performed after removing 104 non-metastasis samples and 100 samples for which Lund subgroup was not available. Of the remaining 260 samples, 260 had survival data, and 135 had recurrence data available. A small group with < 15 samples representing the non-metastatic (Normal or Keratin) subtype was excluded from each analysis, (n=12 for our classification and n=13 for Lund in the survival analysis and n=5 for our classification and n=4 for Lund in the recurrence analysis).

Supplementary Information - Section 5

Validation of the tree on the dataset of Cirenajwis et al., 2015

To validate the three-gene decision tree described in Fig. 4, we downloaded the Lund dataset by Cirenajwis et al.[15] from GEO[17] (GSE65904). The dataset contains expression profiles of 214 stage III melanoma samples, measured by Illumina Human-HT12v4.0 BeadChip arrays. We applied a log2 transformation to the data, and mapped each of the three predictor genes to a corresponding probeset ID in the Lund dataset: A single probeset was available for the TRIM63 gene (ILMN_1702489), where two probesets were available for both TIGIT and KLK8, of which we manually selected one (ILMN_2125017 and ILMN_1735700 respectively).

Before applying the decision tree on the samples, we manually calibrated the tree threshold values. This step was required because the new dataset was generated using a different platform (microarrays) and therefore had different expression value distributions for the three genes compared with the TCGA dataset (RNA-Seq), on which the decision tree was trained. Furthermore, the distributions differed also due to the different characteristics of the samples here compared to TCGA samples, where samples identified as primary had relatively large tumors and thus were likely more advanced[2].

When executed, the decision tree assigned a subgroup label to each one of the melanoma samples (See Table S7). Samples showing high expression of KLK8 (above 7.44) were assigned to the Keratin subgroup (n=13). Out of the remaining samples, those with high expression of TIGIT (above 7.36) were assigned to the Immune subgroup (n=80). Out of the remaining samples, those with high expression of TRIM63 (above 9.45) were assigned to the Melanogenesis-high subgroup (n=41), and lastly, all remaining samples were assigned to the Melanogenesis-low subgroup (See Fig. S16). Remarkably, the Immune, Melanogenesis-low, and Melanogenesis-high subgroups showed distinct survival curves with relative risk that is in agreement with their relative risk on the TCGA dataset. These results demonstrate the ability of the decision tree to identify prognostic subgroups of melanoma (Fig. S17).

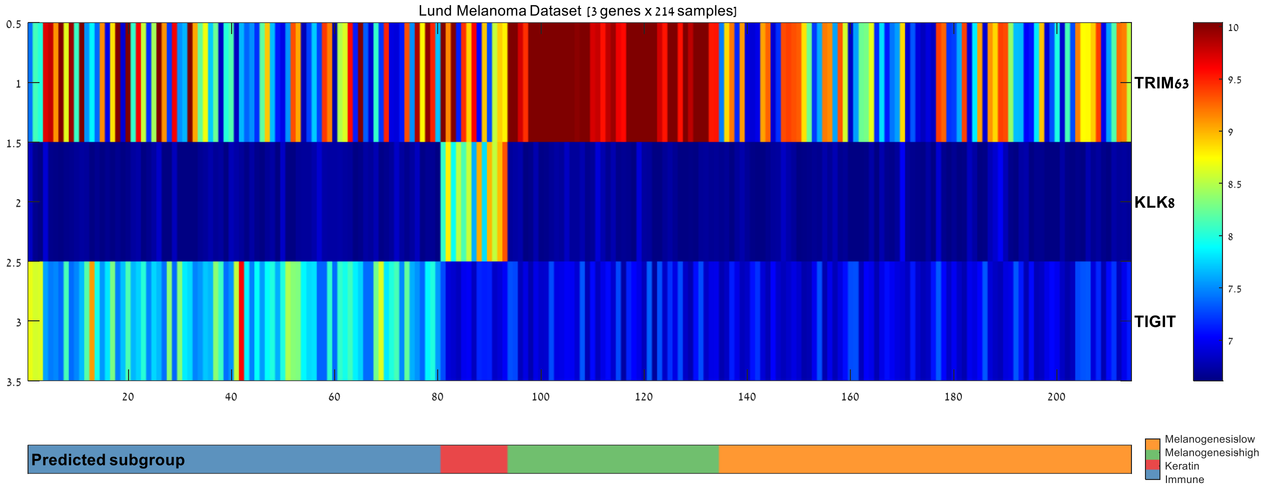

Figure S16: Results of the predictions of the three-gene decision tree on the dataset of Cirenajwis et al. The microarray dataset contained 214 melanoma samples, which were classified by the three-gene decision tree into the four melanoma subgroups: Immune (n=80), Keratin (n=13), Melanogenesis-low (n=80), and Melanogenesis-high (n=41).

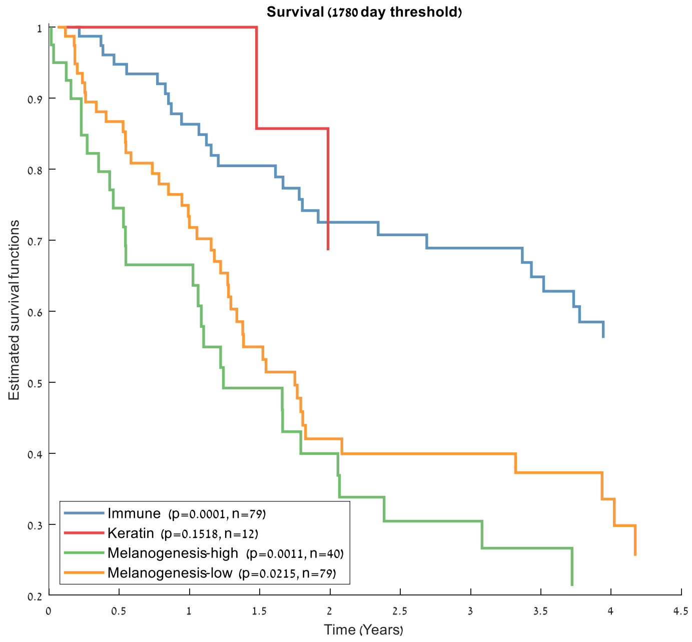

Figure S17: Survival analysis for the four subgroups identified by the three-gene decision tree on the dataset of Cirenajwis et al. The Immune (blue) and Melanogenesis-high (green) subgroups showed significant survival difference compared to the other groups, in agreement with their typical good and bad survival characteristics, respectively, on the TCGA dataset. The small Keratin (red) sample group showed the best survival curve on this dataset, which is what we would expect from a primary enriched subgroup (unlike

this group's worst survival on the TCGA data, which might be attributed to the size bias of the primary tumors in the TCGA melanoma cohort).

Sample Id (GEO)	Call	Sample Id (GEO)	Call	Sample Id (GEO)	Call
GSM1608593	Immune	GSM1608665	Immune	GSM1608737	Melanogenesis-low
GSM1608594	Melanogenesis-low	GSM1608666	Immune	GSM1608738	Immune
GSM1608595	Keratin	GSM1608667	Melanogenesis-low	GSM1608739	Melanogenesis-low
GSM1608596	Immune	GSM1608668	Melanogenesis-high	GSM1608740	Melanogenesis-low
GSM1608597	Melanogenesis-low	GSM1608669	Melanogenesis-high	GSM1608741	Keratin
GSM1608598	Immune	GSM1608670	Immune	GSM1608742	Melanogenesis-low
GSM1608599	Immune	GSM1608671	Immune	GSM1608743	Melanogenesis-low
GSM1608600	Immune	GSM1608672	Melanogenesis-high	GSM1608744	Melanogenesis-high
GSM1608601	Immune	GSM1608673	Keratin	GSM1608745	Immune
GSM1608602	Melanogenesis-low	GSM1608674	Keratin	GSM1608746	Immune
GSM1608603	Keratin	GSM1608675	Melanogenesis-low	GSM1608747	Melanogenesis-low
GSM1608604	Immune	GSM1608676	Melanogenesis-low	GSM1608748	Melanogenesis-low
GSM1608605	Melanogenesis-low	GSM1608677	Immune	GSM1608749	Immune
GSM1608606	Immune	GSM1608678	Keratin	GSM1608750	Immune
GSM1608607	Melanogenesis-low	GSM1608679	Melanogenesis-low	GSM1608751	Melanogenesis-high
GSM1608608	Melanogenesis-low	GSM1608680	Melanogenesis-low	GSM1608752	Melanogenesis-high
GSM1608609	Immune	GSM1608681	Melanogenesis-high	GSM1608753	Immune
GSM1608610	Melanogenesis-low	GSM1608682	Immune	GSM1608754	Immune
GSM1608611	Immune	GSM1608683	Melanogenesis-high	GSM1608755	Immune
GSM1608612	Melanogenesis-high	GSM1608684	Immune	GSM1608756	Melanogenesis-low
GSM1608613	Melanogenesis-low	GSM1608685	Melanogenesis-low	GSM1608757	Melanogenesis-high
GSM1608614	Melanogenesis-low	GSM1608686	Melanogenesis-high	GSM1608758	Melanogenesis-low
GSM1608615	Melanogenesis-low	GSM1608687	Immune	GSM1608759	Melanogenesis-low
GSM1608616	Immune	GSM1608688	Melanogenesis-low	GSM1608760	Melanogenesis-low
GSM1608617	Melanogenesis-high	GSM1608689	Melanogenesis-high	GSM1608761	Melanogenesis-low
GSM1608618	Melanogenesis-low	GSM1608690	Melanogenesis-low	GSM1608762	Melanogenesis-low
GSM1608619	Melanogenesis-low	GSM1608691	Melanogenesis-low	GSM1608763	Immune
GSM1608620	Melanogenesis-low	GSM1608692	Keratin	GSM1608764	Immune
GSM1608621	Melanogenesis-high	GSM1608693	Immune	GSM1608765	Melanogenesis-low
GSM1608622	Melanogenesis-low	GSM1608694	Melanogenesis-high	GSM1608766	Melanogenesis-high
GSM1608623	Immune	GSM1608695	Immune	GSM1608767	Melanogenesis-high
GSM1608624	Melanogenesis-low	GSM1608696	Immune	GSM1608768	Immune
GSM1608625	Immune	GSM1608697	Melanogenesis-low	GSM1608769	Immune
GSM1608626	Immune	GSM1608698	Immune	GSM1608770	Immune
GSM1608627	Melanogenesis-low	GSM1608699	Melanogenesis-high	GSM1608771	Melanogenesis-high
GSM1608628	Melanogenesis-high	GSM1608700	Melanogenesis-low	GSM1608772	Melanogenesis-high
GSM1608629	Melanogenesis-low	GSM1608701	Keratin	GSM1608773	Immune
GSM1608630	Melanogenesis-low	GSM1608702	Melanogenesis-low	GSM1608774	Melanogenesis-low
GSM1608631	Melanogenesis-low	GSM1608703	Immune	GSM1608775	Melanogenesis-low
GSM1608632	Melanogenesis-low	GSM1608704	Melanogenesis-high	GSM1608776	Melanogenesis-high
GSM1608633	Immune	GSM1608705	Immune	GSM1608777	Immune
GSM1608634	Immune	GSM1608706	Keratin	GSM1608778	Melanogenesis-low
GSM1608635	Melanogenesis-high	GSM1608707	Melanogenesis-high	GSM1608779	Immune
GSM1608636	Melanogenesis-low	GSM1608708	Immune	GSM1608780	Melanogenesis-high
GSM1608637	Melanogenesis-high	GSM1608709	Melanogenesis-low	GSM1608781	Immune
GSM1608638	Melanogenesis-low	GSM1608710	Melanogenesis-high	GSM1608782	Immune
GSM1608639	Immune	GSM1608711	Melanogenesis-low	GSM1608783	Melanogenesis-low
GSM1608640	Melanogenesis-high	GSM1608712	Melanogenesis-high	GSM1608784	Melanogenesis-high
GSM1608641	Immune	GSM1608713	Immune	GSM1608785	Melanogenesis-low
GSM1608642	Immune	GSM1608714	Melanogenesis-low	GSM1608786	Immune
GSM1608643	Melanogenesis-low	GSM1608715	Immune	GSM1608787	Keratin
GSM1608644	Melanogenesis-low	GSM1608716	Melanogenesis-low	GSM1608788	Melanogenesis-low
GSM1608645	Melanogenesis-high	GSM1608717	Melanogenesis-high	GSM1608789	Melanogenesis-high
GSM1608646	Melanogenesis-low	GSM1608718	Melanogenesis-low	GSM1608790	Melanogenesis-high
GSM1608647	Melanogenesis-low	GSM1608719	Immune	GSM1608791	Immune
GSM1608648	Immune	GSM1608720	Immune	GSM1608792	Immune
GSM1608649	Melanogenesis-high	GSM1608721	Melanogenesis-low	GSM1608793	Immune
GSM1608650	Melanogenesis-low	GSM1608722	Immune	GSM1608794	Immune
GSM1608651	Melanogenesis-low	GSM1608723	Melanogenesis-low	GSM1608795	Immune
GSM1608652	Immune	GSM1608724	Melanogenesis-low	GSM1608796	Immune
GSM1608653	Immune	GSM1608725	Immune	GSM1608797	Keratin
GSM1608654	Keratin	GSM1608726	Melanogenesis-low	GSM1608798	Immune
GSM1608655	Melanogenesis-low	GSM1608727	Immune	GSM1608799	Immune
GSM1608656	Immune	GSM1608728	Melanogenesis-low	GSM1608800	Melanogenesis-high
GSM1608657	Immune	GSM1608729	Melanogenesis-low	GSM1608801	Immune
GSM1608658	Immune	GSM1608730	Keratin	GSM1608802	Melanogenesis-high
GSM1608659	Melanogenesis-low	GSM1608731	Immune	GSM1608803	Melanogenesis-low
GSM1608660	Melanogenesis-low	GSM1608732	Immune	GSM1608804	Melanogenesis-low
GSM1608661	Melanogenesis-high	GSM1608733	Melanogenesis-low	GSM1608805	Immune
GSM1608662	Immune	GSM1608734	Melanogenesis-low	GSM1608806	Immune
GSM1608663	Melanogenesis-low	GSM1608735	Melanogenesis-high		
GSM1608664	Melanogenesis-high	GSM1608736	Melanogenesis-low		

Table S7: Predicted melanoma subgroup for the Lund dataset samples as called by three gene classifier.

Acknowledgments

The results published here are based upon data generated by The Cancer Genome Atlas managed by the NCI and NHGRI. Information about TCGA can be found at http://cancergenome.nih.gov.

The Genotype-Tissue Expression (GTEx) Project was supported by the Common Fund of the Office of the Director of the National Institutes of Health, and by NCI, NHGRI, NHLBI, NIDA, NIMH, and NINDS.

Figure S7 was generated by the Human Protein Atlas, available from http://www.proteinatlas.org.

References:

1. Netanely, D., Stern, N., Laufer, I. & Shamir, R. PROMO: an interactive tool for analyzing clinically-labeled multi-omic cancer datasets. *BMC Bioinformatics* **20**, 732 (2019).
2. Akbani, R. *et al.* Genomic Classification of Cutaneous Melanoma. *Cell* **161**, 1681–1696 (2015).
3. Shamir, R. *et al.* EXPANDER--an integrative program suite for microarray data analysis. *BMC Bioinformatics* **6**, 232 (2005).
4. Ulitsky, I. *et al.* Expander: from expression microarrays to networks and functions. *Nat. Protoc.* **5**, 303–22 (2010).
5. Kanehisa, M. & Goto, S. KEGG: kyoto encyclopedia of genes and genomes. *Nucleic Acids Res.* **28**, 27–30 (2000).
6. Uhlén, M. *et al.* Tissue-based map of the human proteome. *Science (80-.).* (2015) doi:10.1126/science.1260419.
7. The Human Protein Atlas. https://www.proteinatlas.org/.
8. The Genotype-Tissue Expression (GTEx) project. https://www.gtexportal.org/home/.
9. The Functional Annotation of Mammalian Genomes 5. https://fantom.gsc.riken.jp/5/.
10. Lizio, M. *et al.* Gateways to the FANTOM5 promoter level mammalian expression atlas. *Genome Biol.* (2015) doi:10.1186/s13059-014-0560-6.
11. Lizio, M. *et al.* Update of the FANTOM web resource: Expansion to provide additional transcriptome atlases. *Nucleic Acids Res.* (2019) doi:10.1093/nar/gky1099.
12. Eden, E., Navon, R., Steinfeld, I., Lipson, D. & Yakhini, Z. GOrilla: a tool for discovery and visualization of enriched GO terms in ranked gene lists. *BMC Bioinformatics* **10**, 48 (2009).
13. Jönsson, G. *et al.* Gene expression profiling-based identification of molecular subtypes in stage IV melanomas with different clinical outcome. *Clin. Cancer Res.* **16**, 3356–67 (2010).
14. Nsengimana, J. *et al.* Independent replication of a melanoma subtype gene signature and evaluation of its prognostic value and biological correlates in a population cohort. *Oncotarget* **6**, 11683–93 (2015).
15. Cirenajwis, H. *et al.* Molecular stratification of metastatic melanoma using gene expression

profiling : Prediction of survival outcome and benefit from molecular targeted therapy. *Oncotarget* **6**, 12297–12309 (2015).

16. Lauss, M., Nsengimana, J., Staaf, J., Newton-Bishop, J. & Jönsson, G. Consensus of Melanoma Gene Expression Subtypes Converges on Biological Entities. *J. Invest. Dermatol.* **136**, 2502–2505 (2016).

17. Edgar, R., Domrachev, M. & Lash, A. E. Gene Expression Omnibus: NCBI gene expression and hybridization array data repository. *Nucleic Acids Res.* **30**, 207–10 (2002).
